# Beyond the Walls of Troy: A Scoping Review on Pharmacological Strategies to Enhance Drug Delivery Across the Blood–Brain Barrier and Blood–Tumor Barrier

**DOI:** 10.3390/ijms26157050

**Published:** 2025-07-22

**Authors:** Miłosz Pinkiewicz, Artur Zaczyński, Jerzy Walecki, Michał Zawadzki

**Affiliations:** 1 Department of Neurosurgery, The National Medical Institute of the Ministry of the Interior and Administration, 02-507 Warsaw, Poland; 2 Division of Interventional Neuroradiology, Department of Radiology, The National Medical Institute of the Ministry of the Interior and Administration, 02-507 Warsaw, Poland; 3 Department of Radiology Warszawa, The National Medical Institute of the Ministry of the Interior and Administration, 02-507 Warsaw, Poland

**Keywords:** blood–brain barrier, blood–tumor barrier, bradykinin receptor, adenosine receptor, efflux inhibitors, BBB disruption, tumor microenvironment, passive targeting, active targeting, Trojan Horse strategy

## Abstract

The blood–brain barrier (BBB) is a highly selective interface between the bloodstream and the brain that prevents systemically administered therapeutics from effectively reaching tumor cells. As tumors progress, this barrier undergoes structural and functional alterations, giving rise to the blood–tumor barrier (BTB)—a pathologically modified structure that, despite increased permeability, often exhibits heterogeneous and clinically insufficient drug transport. Although a new generation of therapies is promising, their therapeutic potential cannot be realized unless the challenges posed by these barriers are effectively addressed. Various pharmacological strategies were explored to enhance brain tumor drug delivery. These include receptor-mediated disruption, inhibition of efflux transporters, and the engineering of delivery platforms that leverage endogenous transport pathways—such as carrier-mediated, adsorptive-mediated, and receptor-mediated mechanisms—as well as cell-mediated drug delivery. This review synthesizes (1) the BBB and BTB’s structural characteristics; (2) the influence of the tumor microenvironment (TME) on drug delivery; (3) pharmacological strategies to enhance drug accumulation within brain tumors; (4) the integration of pharmacological methods with neurosurgical techniques to enhance drug delivery. As efforts to improve drug delivery across the BBB and BTB accelerate, this review aims to map the current landscape of pharmacological approaches for enhancing drug penetration into brain tumors.

## 1. Introduction

Although constituting only 2% of all cancers, brain tumors rank among the most formidable malignancies, with glioblastoma multiforme (GBM) being the most common malignant primary brain tumor [1]. The current standard of care entails maximal safe surgical resection followed by adjuvant chemoradiotherapy with temozolomide (TMZ). Despite this multimodal approach, median overall survival remains dismal at approximately 15–18 months, although patients who undergo gross total resection (GTR) may achieve prolonged survival of up to 20–25 months [2].

While each therapeutic modality offers incremental benefit, significant limitations persist. Complete surgical resection is frequently unachievable due to the tumor’s diffuse infiltration, migration along white matter tracts, multifocal presentation, or proximity to eloquent brain regions. Moreover, many brain tumors develop marked radioresistance through molecular and cellular adaptations, including the upregulation of DNA damage response proteins and aberrant activation of pro-survival pathways such as phosphoinositide 3-kinase (PI3K)/protein kinase B (Akt), mitogen-activated protein kinase (MAPK), and nuclear factor kappa-light-chain-enhancer of activated B cells (NF-κB) [3]. The hypoxic tumor microenvironment (TME) further compromises the efficacy of radiotherapy by limiting the availability of oxygen, a critical radiosensitizer, thereby facilitating tumor cell survival and metabolic adaptation [3].

Finally, pharmacologic therapies have yielded only modest improvements in survival, largely due to two principal challenges: the substantial genetic and molecular heterogeneity of brain tumors, which contribute to therapeutic resistance by enabling distinct subpopulations of tumor cells to evade targeted treatments and adapt to selective pressures imposed by therapy, and the restricted permeability of the blood–brain barrier (BBB), which significantly limits the delivery of therapeutic agents to the tumor site [4,5]. The latter remains arguably the most critical—yet frequently under-recognized—obstacle to therapeutic success. Emerging strategies such as molecularly targeted agents, personalized therapies, immunomodulators, and multi-agent regimens aim to address the biological complexity of brain tumors [5,6]. However, their success remains fundamentally constrained by the pharmacokinetic bottleneck imposed by the BBB. To fulfill their therapeutic potential, brain tumor treatments must not only engage relevant oncogenic targets, but also effectively cross the BBB, accumulate at cytotoxic levels within tumor cells, and overcome the physical and metabolic defenses of the TME [7]. Without sufficient drug accumulation in tumor cells, even the most rationally designed therapies are unlikely to yield meaningful clinical benefit—a reality reflected in the underwhelming outcomes of recent clinical trials [8,9].

Formed by a monolayer of microvascular endothelial cells lining cerebral vessels, the BBB serves as a tightly regulated interface between the central nervous system (CNS) and the bloodstream, protecting the brain from pathogens while ensuring the controlled transport of essential molecules [7]. Molecular transport across the BBB primarily occurs through transcellular mechanisms, as tight junctions (TJs) between brain endothelial cells (bECs) prevent paracellular diffusion, with exceptions for small, gaseous molecules (e.g., O_2_ and CO_2_) and highly lipophilic compounds (e.g., ethanol, barbiturates) [10]. Passive diffusion is limited to molecules that meet specific physicochemical criteria, including a molecular weight ≤ 450 Da, a lipophilic nature, and minimal hydrogen bonding (<8 hydrogen bonds) [11]. However, to date, no approved brain tumor drug fully meets the physicochemical criteria for passive diffusion across the BBB. In addition, the BBB expresses various regulated efflux transporters, most notably P-glycoprotein (P-gp/ABCB1) and breast cancer resistance protein (BCRP/ABCG2). These actively prevent access to relatively lipophilic compounds that have some degree of passive permeability to the endothelial lining of the brain microvasculature [7]. For instance, TMZ, a mainstay of GBM treatment, achieves only 20–30% of its plasma concentration in the CNS [12].

Large molecules—including peptides, recombinant proteins, monoclonal antibodies, RNA interference (RNAi)-based drugs, and gene therapies—are unable to cross the BBB unaided due to their size and hydrophilicity. They require specialized transport mechanisms such as carrier-mediated transport (CMT), receptor-mediated transcytosis (RMT), or adsorptive-mediated transcytosis (AMT) [11]. Even when engineered to exploit these pathways, macromolecules encounter additional formidable hurdles, including enzymatic degradation by circulating peptidases, proteases, and nucleases; rapid clearance by the reticuloendothelial system (RES); and intracellular sequestration or degradation following endocytosis [11]. Moreover, once past the BBB, the brain’s extracellular space (ECS) presents a dense, viscous matrix of proteoglycans and hyaluronic acid. This effectively acts as a “molecular sieve” for large molecules, significantly impeding their distribution and penetration into deeper brain tissues. Collectively, these elaborate protective mechanisms ensure that over 98% of small-molecule drugs—and nearly all large-molecule therapeutics—fail to reach the brain parenchyma at therapeutically effective concentrations and exert their intended therapeutic effects.

As primary and metastatic brain tumors develop, they disrupt the BBB, forming the blood–tumor barrier (BTB) [7]. However, despite a ten-fold increase in permeability in tumor-associated capillaries, the BTB remains 100 to 100,000 times less permeable to polar solutes than peripheral capillaries. Moreover, BBB disruption is highly variable, leading to heterogeneous drug delivery [7,13]. For example, brain-to-plasma Cmax ratios for TMZ can range from 1.9% to 24.6%, highlighting the uneven distribution of therapeutics within tumors [14]. Furthermore, large portions of the tumor can remain shielded by an intact BBB, protecting aggressive cancer stem cells from the tumor agent. Considering these factors, as well as irregular blood flow and active efflux transporters, the BTB represents a major obstacle to effective drug delivery [7,15].

The BBB and BTB pose formidable obstacles to effective drug delivery in primary and metastatic brain tumors, contributing significantly to the limited success of current therapies. This underscores the urgent need for innovative strategies that can bypass these barriers and optimize drug delivery, ultimately improving therapeutic outcomes in neuro-oncology. Advances in our understanding of the BBB and BTB —much of which has emerged from preclinical studies in rodent models—along with insights into the factors contributing to their impermeability and the cellular mechanisms governing transport, have paved the way for the development of pharmacological strategies aimed at increasing the concentration of systemically administered drugs within the brain. Herein, we comprehensively review BBB and BTB structure, and the TME from the perspective of drug delivery, and deliver an overview of pharmacological strategies aimed at improving drug concentrations within tumors—including receptor-mediated disruption, efflux transporter inhibitors, and targeted transport mechanisms (RMT, CMT, AMT). Additionally, we discuss how specific pharmacological strategies can be integrated with neurointerventional and neurosurgical techniques to further optimize drug delivery to brain tumors.

## 2. Methods

This scoping review was conducted in accordance with the PRISMA Extension for Scoping Reviews (PRISMA-ScR) guidelines. A structured literature search was performed in April 2025 across the PubMed/MEDLINE and Cochrane Library databases. The search included English-language articles published from database inception through the date of search. Search terms included combinations of “blood–brain barrier”, “blood–tumor barrier”, “bradykinin receptor”, “adenosine receptor”, “efflux inhibitors”, “efflux pumps”, “BBB disruption”, “tumor microenvironment”, “passive targeting”, “active targeting”, “carrier-mediated delivery”, “cell-mediated delivery”, “Trojan Horse strategy”, “receptor-mediated delivery”, “adsorptive-mediated delivery”, “BBB drug delivery”, “osmotic BBB disruption”, “focused ultrasound”, and “convection-enhanced delivery.” The review protocol was not registered.

A two-stage screening process was employed. First, four independent reviewers (M. Pinkiewicz, A. Zaczyński, J. Walecki, and M. Zawadzki) screened titles and abstracts for relevance. Full-text articles were then assessed for eligibility. Studies were included if they were peer-reviewed, available as full texts, and focused on either the structure or function of the BBB or BTB, TME’s influence on drug delivery, or on pharmacological strategies aimed at overcoming BBB/BTB in the context of brain tumor therapy. Both preclinical and clinical studies were considered eligible.

Exclusion criteria included non-peer-reviewed content, incomplete data, and insufficient methodological transparency. Poor methodological transparency was defined as a lack of essential study details, including (i) clearly stated objectives or hypotheses, (ii) descriptions of drug dosing or delivery protocols, (iii) pharmacokinetic or drug distribution endpoints (e.g., brain-to-plasma ratios), (iv) specification of study controls, replication strategy, or statistical analysis. Studies that did not report the experimental model used (in preclinical studies) or failed to describe trial phase and population (in clinical studies) were also excluded.

Data extraction was conducted independently and reviewed in duplicate. Discrepancies were resolved by consensus among all reviewers. Risk of bias was assessed by two reviewers and verified by two others.

## 3. Results

**Figure 1:**
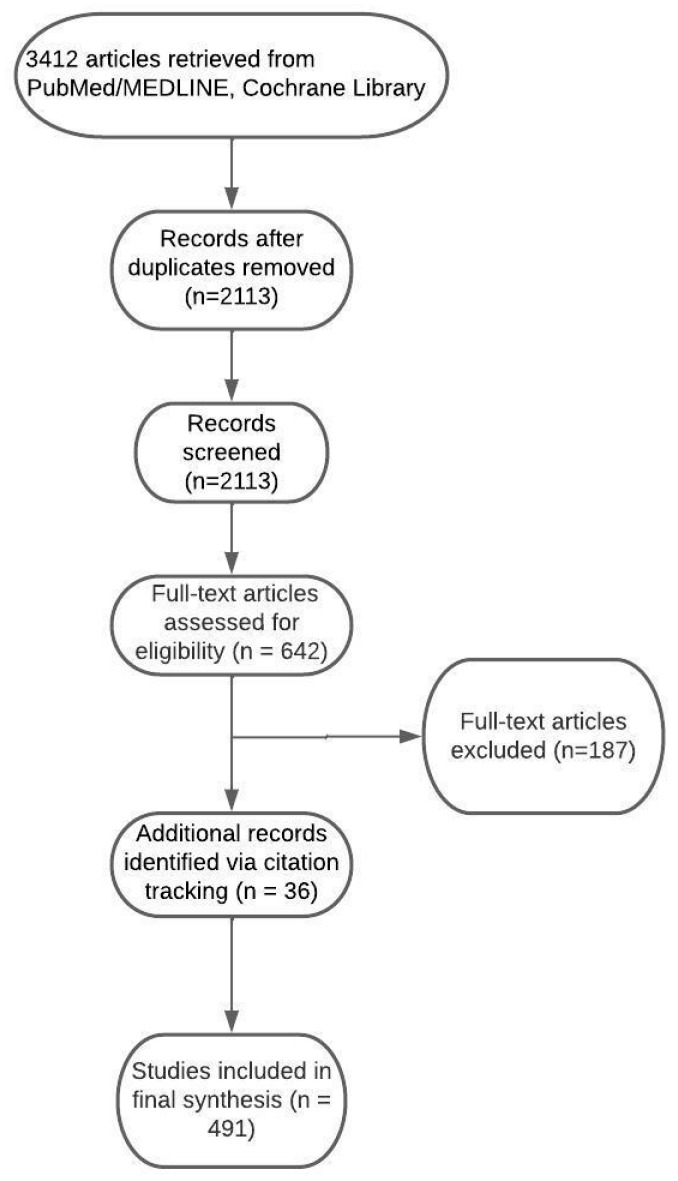
Flow diagram demonstrating process of article selection.

The initial database search yielded 3412 records. After removing duplicates, 2113 articles remained for title and abstract screening. Of these, 642 articles were selected for full-text evaluation. In total, 187 articles were excluded based on language, design, or methodological quality. An additional 36 records were identified through citation tracking. In total, 491 articles were included in the final synthesis. The study selection process is illustrated in Figure 1.

### 3.1. Blood–Brain Barrier

Three barriers govern molecular exchange between the blood and the CNS, preserving its stability and function: (i) the BBB, which separates the blood from the brain interstitial fluid (ISF), and is formed by endothelial cells sealed by continuous TJs and adherens junctions (AJs), supported by pericytes, a shared basement membrane (BM), and astrocytic endfeet; (ii) the choroid plexus epithelium, which separates blood from ventricular cerebrospinal fluid (CSF); (iii) the arachnoid epithelium, which lies between the blood and subarachnoid CSF [16]. Among these, the BBB plays the most crucial role in maintaining the brain’s microenvironment and serves as the primary site of blood–CNS exchange [16,17]. This dominant role is attributed to two factors: first, the proximity of neurons, which are located just 8–20 µm from a brain capillary, compared to their distance from CSF compartments, which can be millimeters or even centimeters [18]. Second, the BBB is present at all levels of the vascular tree within the CNS, including penetrating arteries, arterioles, dense capillary beds, post-capillary venules, and draining veins. This widespread distribution results in a substantial total exchange area of 12 to 18 m^2^ in the average adult brain [11]. However, the BBB is not uniform across all brain regions. In circumventricular organs (CVOs)—such as the pineal gland, median eminence, neurohypophysis, and area postrema—blood vessels exhibit increased permeability due to structural differences in bECs and perivascular interactions, enabling specialized neurohumoral functions [19,20].

Our understanding of the BBB has evolved from a static, impermeable wall to a dynamic, highly selective system influenced by local and circulating factors [7]. Microglia—the predominant innate immune cells in the CNS—can both disrupt and repair the BBB. During inflammation, microglia and peripheral immune cells, such as leukocytes, increase BBB permeability by secreting interleukin-1β (IL-1β) or expressing adhesion molecules like ICAM1, VCAM1, and E-selectin [7,21]. Additionally, the BBB is innervated by synaptic endings, where GABAergic, cholinergic, noradrenergic, and serotonergic neurons directly modulate endothelial function and blood flow [22].

**Figure 2:**
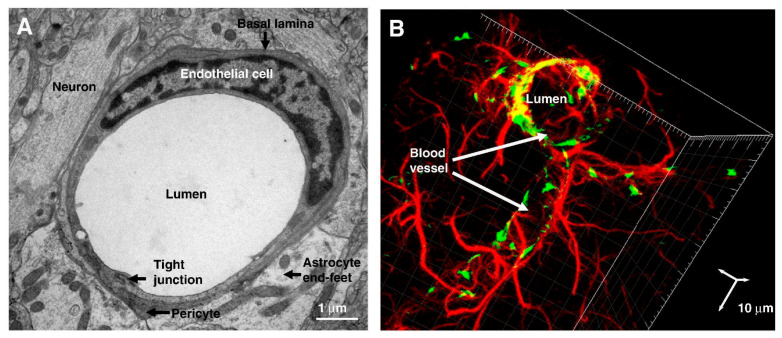
The neurovascular unit. Reused from Weiss et al. [21]. (**A**) A transmission electron microscopy (TEM) image of a rat brain section depicting the ultrastructure of an NVU. The microvessel is composed of endothelial cells surrounded by a continuous basal lamina, with pericytes embedded within it. Astrocytic end-feet envelop the vessel, and adjacent neuronal structures are visible in the surrounding parenchyma. (**B**) A three-dimensional confocal reconstruction of a rat brain section showing part of the cerebral vascular network. Endothelial cells (green), labeled with von Willebrand factor, are surrounded by astrocytes (red), visualized using glial fibrillary acidic protein (GFAP) staining.

The BBB exhibits remarkable adaptability, driven by the coordinated interactions between bECs, mural cells (pericytes and vascular smooth muscle cells, VSMCs), astrocytes, neurons, and microglia, all of which share a common basement membrane. Together, these components constitute the neurovascular unit (NVU), a highly regulated structure that controls molecular and cellular exchanges within the CNS (Figure 2) [23]. The NVU model has evolved from a simplistic view of neuronal–astrocytic signaling to a more complex network, where diverse cellular elements engage in coordinated but distinct pathways [23]. As the anatomical and functional core of the NVU, the BBB not only regulates barrier integrity and maintains the perivascular microenvironment but also controls cerebral blood flow and orchestrates responses to brain injury.

### 3.2. Endothelial Cells

Endothelial cells lining the cerebral vessels are the core component of the BBB, tightly connected by both TJs and AJs. On the luminal side, bECs are coated with a glycocalyx layer, which forms a negatively charged shield around surface adhesion molecules, restricting the passage or interaction of blood-borne cells (such as immune and tumor cells) and large molecules [23,24]. On the abluminal (brain-facing) side, bECs are encased by pericytes, which are embedded within a shared BM. Surrounding this structure are the astrocytic end-feet, which form crucial cellular connections with neurons, contributing to the overall integrity and function of the BBB [24].

Several unique characteristics of these bECs contribute to their selective permeability [11]. First, they lack fenestrations, i.e., small transcellular pores that restrict free diffusion and the rapid exchange of molecules between the blood and the CNS [25,26]. Additionally, their net negative surface charge repels negatively charged compounds, and the low expression of leukocyte adhesion molecules limits the entry of various immune cells [25]. Furthermore, the high transendothelial electrical resistance across these cells reduces the formation of vesicles, restricting the transcellular transport of molecules across the vessel wall [25,26,27]. Finally, bECs express a wide array of specific transporter systems for regulating the inflow and outflow of substrates, which will be discussed in detail later in the review, as many of these transporters serve as key targets for enhancing drug delivery to the CNS [28].

### 3.3. Basal Membrane

The BM consists of two primary layers: an inner vascular basement membrane, secreted by bECs and pericytes, and an outer parenchymal boundary membrane called the membrana limitans gliae perivascularis (MLGP), formed by astrocyte end-feet processes [11]. The BM provides structural support and facilitates extracellular signaling among cells of the NVU while also creating an additional physical barrier that limits the migration of cells and molecules into the CNS. Under healthy conditions, these two BM layers appear indistinguishable, though a potential perivascular space exists between them [29]. In pathological states, this space can serve as a checkpoint where leukocytes accumulate, regulating their further passage into the brain’s parenchyma [7,29].

### 3.4. Pericytes

Enveloping nearly 100% of the microvascular length and approximately 37% of the vessel circumference, the CNS exhibits the highest pericyte coverage among all vascularized tissues [30]. As central components of the NVU, they play a crucial role in regulating BBB integrity, permeability, and function [31]. Pericytes also modulate angiogenesis and vascular remodeling through vascular endothelial growth factor (VEGF) and Notch signaling [32,33]. Additionally, they respond to cytokines and inflammatory mediators, regulating the infiltration of peripheral immune cells into the CNS and contributing to neuroinflammation [32,33].

Loss of pericytes, due to aging or disease, is linked to BBB breakdown, neuroinflammation, and neurodegeneration. Pericytes are essential for BBB development, producing components of the basal lamina and secreting signaling factors that regulate BBB-specific gene expression in bECs and induce astrocyte end-foot polarization [34,35,36,37]. They also promote TJ formation and vesicle trafficking in bECs [38]. Furthermore, pericytes secrete vitronectin, an extracellular matrix (ECM) protein that binds to the integrin receptor α5 in bECs, inhibiting transcytosis [38]. As key regulators of BBB integrity and capillary blood flow, pericytes have significant implications for CNS drug delivery [39]. The passage of molecules into the brain parenchyma, measured as the amount of substance per unit gram of brain tissue, depends on both the permeability characteristics of the BBB and the available surface area for exchange, which together determine the permeability surface product [40]. Alterations in cerebral blood flow have minimal impact on the absolute amount of circulating substances entering the brain when those substances have poor BBB permeability [40]. However, if a substance is highly permeable by nature or if the BBB becomes more permeable due to pathology, cerebral blood flow becomes a key determinant [40]. Thus, by regulating both the surface area of the BBB and cerebral blood flow, pericytes modulate barrier function in a region-specific manner. Given their ability to regulate transcytosis, maintain BBB integrity, and modulate cerebral blood flow, pericytes are central to determining the extent and regional specificity of drug permeability into the CNS. 

### 3.5. Astrocytes

Astrocytes are the most abundant glial cells in the CNS, forming extensive networks where individual cells are interconnected by gap junctions that enable intercellular communication [41]. The abluminal sides of CNS blood vessels are fully enveloped by astrocyte end-feet, which connect to the BM via the dystroglycan–dystrophin complex and the proteoglycan agrin [41]. Nearly all gray matter astrocytes are connected to at least one blood vessel, with a higher density of vascular contacts observed in deeper cortical layers, where vessel density is greater [42]. On the opposite side, astrocytic end-feet also cover synapses, where they sense neuronal activity, modulate synaptic transmission by synthesizing and clearing neurotransmitters, and regulate the local ionic environment via gliotransmission [42]. 

Located at the interface between blood vessels and neurons within the NVU, astrocytes are strategically positioned to regulate vascular tone [43]. Through the secretion of vasoactive molecules from their end-feet onto underlying VSMCs, they influence vascular tone in a process known as functional hyperemia or neurovascular coupling (NVC), adjusting cerebral blood flow to meet the energy demands of active neurons [44]. Additionally, astrocytes engage in dynamic signaling within the NVU, helping regulate water content in the neuroparenchyma through the water channel protein aquaporin 4 (AQP4) [44]. They also mediate angiogenesis, contribute to the production of specialized enzyme systems, and, together with pericytes, enable the polarization of transporters [44].

Astrocytes are believed to play an essential role in maintaining BBB integrity under both normal and pathological conditions [45]. However, the precise cellular mechanisms behind this function remain unclear. A recent study showed that a subset of astrocytes expressing dentin matrix acidic phosphoprotein 1 (Dmp1) helps maintain BBB integrity and angiogenic function by transferring mitochondria to bECs. Deletion of the Mitofusin 2 (Mfn2) gene in Dmp1-expressing astrocytes inhibited mitochondrial transfer and led to BBB leakage [46]. Interestingly, a study using laser ablation to remove astrocytic end-feet covering blood vessels in the in vivo mouse brain found that the BBB remained intact, with no leakage of plasma marker dyes such as Evans Blue or fluorescein–dextran [47]. These findings suggest that astrocytes may contribute to BBB maintenance in a more dynamic or indirect manner, potentially involving mechanisms beyond their direct physical association with blood vessels.

### 3.6. Tight Junctions

TJs, also known as occluding junctions or zonula occludens, form a crucial network of protein strands on the lateral membrane of bECs, sealing the interendothelial cleft and regulating paracellular transport of ions, water, and macromolecules [48,49]. At the BBB, TJs reside predominantly on the protoplasmic face (P-face) of the membrane, unlike peripheral endothelial cells, where they are located on the extracellular face [48,49,50].

TJs are formed by transmembrane proteins, including claudins (Cldns); TJ-associated marvel proteins (TAMPs) such as occludin, marvelD3, and tricellulin; junctional adhesion molecules (JAMs) including JAM-A, JAM-B, and JAM-C; and endothelial-cell-selective adhesion molecules (ESAMs), newly identified transmembrane junction proteins with a similar structure to JAM proteins [48,49,50,51,52]. These proteins interact with their counterparts on adjacent cells and are anchored to the cytoskeleton through actin-binding proteins like ZO-1 and ZO-2, which also play roles in intracellular signaling [49,51]. JAM-A regulates TJs formation and leukocyte adhesion, while ESAM is essential for endothelial cell–cell interactions and neutrophil extravasation during early inflammation [53].

Occludin, first identified as a TJ protein, is commonly used as a marker of BBB integrity [54]. While numerous in vitro studies suggest its involvement in maintaining BBB function, its precise role in vivo and its contribution to overall BBB integrity are still not fully understood [55].

Cldns are the primary sealing components of TJs and regulate paracellular permeability. Cldns exhibit tissue-specific expression, with some being constitutively expressed, while others are inducible in response to specific stimuli or conditions. Cldn-5 is the principal TJ protein at the BBB [56,57,58], with mRNA expression levels nearly 1000 times greater than Cldn-1, -3, and -12 [56]. A study demonstrated that Cldn-5 knockout in mice does not cause overall TJ morphological disruption but selectively increases paracellular permeability to molecules <  800 Da [57]. The fact that Cldn-5-deficient mice succumbed to brain edema within 10 h of birth suggests that other Cldns cannot compensate for Cldn-5 to maintain BBB integrity [49]. 

Ongoing research continues to explore the roles of other Cldns within the BBB [59]. Recent studies suggest that Cldn-11 plays a significant role in TJ formation at the BBB, comparable to Cldn-5 [60]. In contrast, Cldn-1, which was previously thought to be involved in BBB formation [61], is absent from CNS parenchymal microvessels as well as from primary human and mouse bECs, as confirmed by single-cell RNA sequencing (scRNAseq) [62]. Although Cldn-3 has been hypothesized to contribute to BBB development [63], its presence in BBB endothelium has been refuted by multiple methods, including immunostaining, Western blotting, and scRNAseq analysis [64]. While Cldn-12 is detectable in the BBB, its expression remains low, and its absence does not affect barrier integrity [65].

### 3.7. Adherens Junctions

AJs play a pivotal role in maintaining BBB integrity through dynamic interplay with TJs. While TJs establish the primary paracellular barrier, AJs reinforce this function by organizing TJ proteins and stabilizing intercellular adhesion [4,66,67]. In bECs, vascular endothelial (VE)-cadherin is the predominant AJ transmembrane protein, with minimal expression of N- and E-cadherins [68]. VE-cadherin interacts with cytoplasmic proteins—β-catenin, α-catenin, p120, and plakoglobin—to tether the junctional complex to the actin cytoskeleton and modulate outside-in signaling [67,69]. This complex also associates with ZO-1, providing structural and functional integration between AJs and TJs [4,66].

Beyond its structural role, VE-cadherin regulates the transcription of Cldn-5, linking junctional architecture to gene expression [70,71]. This regulation involves the inhibition of FoxO1 and β-catenin nuclear accumulation, which otherwise repress Cldn-5 transcription [70,71]. β-catenin serves a dual function: as a structural component anchoring AJs to the cytoskeleton and as a transcriptional effector in the Wnt signaling pathway [71,72,73]. Although Wnt/β-catenin signaling is less active in adult endothelium, it remains crucial for maintaining BBB integrity by modulating TJ components and controlling transcellular transport [72,73,74]. Inflammatory stimuli can suppress this pathway, leading to barrier dysfunction and increased CNS vulnerability [75].

### 3.8. Blood–Tumor Barrier in Primary Tumors

**Figure 3:**
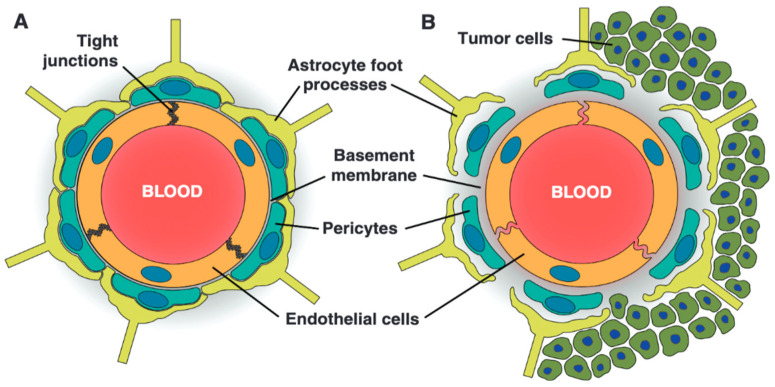
The structural differences between the BBB and BTB (reused from Blethen et al. [83]). (**A**) The BBB is composed of a monolayer of non-fenestrated bECs joined by TJs, supported by pericytes and astrocytic end-feet, all embedded within an intact BM. (**B**) The BTB exhibits pathological alterations induced by tumor progression, including endothelial fenestrations, a disrupted basement membrane, the detachment of pericytes and astrocytes, and elevated pinocytic activity—resulting in heterogeneous and often impaired barrier function.

For many years, the BTB remained conceptually under-recognized—regarded not as a distinct physiological structure but as a dysfunctional extension of the BBB that emerged in the setting of brain tumors [7]. However, research has since revealed that the BTB is a structurally and functionally distinct entity (Figure 3) [76,77]. Invading tumor cells disrupt the perivascular niche, leading to increased vascular permeability and dysfunction of the BTB. Moreover, targeted proteomic analyses of microvessels isolated from human GBM specimens revealed significantly decreased protein levels of both P-gp/ABCB1 and BCRP/ABCG2 compared with normal brain microvessels [76].

Given that interactions between cancer cells and blood vessels occur at varying levels, the resulting structural alterations and functional adaptations across the tumor’s vascular network are highly heterogeneous, a hallmark of the BTB [7,77,78]. Consequently, the degree of BTB permeability varies widely across and within tumors and depends on tumor subtype, grade, and local microenvironment. For instance, the four molecular subtypes of medulloblastoma display differing degrees of endothelial cell fenestration: the WNT subtype has a notably fenestrated vasculature, while the SHH subtype retains an intact BBB [7,79]. Similarly, GBMs have different regions of BTB integrity—bulky tumors are characterized by a dysfunctional BTB, and less invasive circumferential regions have a leaking BTB, whereas areas far from the tumor bulk can display a fully functional BTB [80]. These distant tumor cells extending beyond the primary tumor mass and the surrounding edema depicted by the fluid-attenuated inversion recovery (FLAIR) sequence in magnetic resonance imaging (MRI) scan have intact BBBs and harbor invasive glioblastoma stem cells (GSCs), which, due to significant levels of transcriptional variability and metabolic flexibility, demonstrate remarkable adaptability properties, making them a primary driver of GBM recurrence and treatment resistance [81].

Primary tumors generally have a greater impact on BBB structure and function than metastatic brain cancers. This is due to their location within brain tissue, their infiltrative growth pattern, and their ability to physically displace astrocytic end-feet [82]. As tumors progress, they secrete a range of molecular signals that disrupt endothelial TJs through downregulation of Cldn-5 and occludin, displace astrocytic processes, remodel the extravascular BM, and recruit diverse pericyte populations [82,83,84,85]. Moreover, the upregulation of aquaporin IV in GBMs and astrocytomas leads to a loss of polarized distribution in the end-feet of high-grade tumors, potentially explaining the migration capability of the astrocytoma [86]. The elevated metabolic demand of tumors induces local hypoxia and the heightened expression of hypoxia-inducible factor-1 (HIF-1). Hypoxia not only supports the survival of neural and GSCs, which are inherently drug-resistant and possess tumorigenic potential, but it also stimulates the production of VEGF, leading to the formation of abnormal, leaky, and poorly organized tumor blood vessels, with an overexpression of transporters and receptors to meet the high metabolic demands of the rapidly proliferating tumor cells. Gliomas can acquire their blood supply through various vascularization mechanisms beyond classical angiogenesis that contribute to the heterogenous vasculature and challenging delivery of therapeutics [87,88]. One such mechanism is vasculogenic mimicry (VM), where tumor cells form vessel-like structures independently of bECs. This process can contribute to tumor growth and may play a role in resistance to anti-angiogenic therapies [87,88]. Another mechanism, tumor-to-endothelial transdifferentiation (TDEC), involves GBM cells, including GSCs, transforming into bECs, thereby directly contributing to the formation of new blood vessels [89]. This process contributes to the formation of the perivascular niche (PVN), a specialized microenvironment within GBM that supports GSC survival and proliferation. GSCs and the BBB’s endothelium have a reciprocal interaction that creates a self-sustaining microenvironment where the endothelial cells support the growth and stemness of GSCs, and GSCs, in turn, drive the angiogenesis that sustains the glioma’s rapid expansion [87,88,89]. However, it remains unclear whether GBM cells can directly influence or contribute to the BTB’s integrity and drug resistance without transforming into these vascular cell types, as is the case with medulloblastoma cells that directly contribute to BTB formation and function.

These intricate mechanisms underscore the extensive vascularization capacity of GBMs. In contrast, metastatic lesions often display a significantly lower vascular density, typically only 12–15% of a GBM, reflecting their differing abilities to induce new vessel formation [90]. In addition, GBM vessels are more compromised. In preclinical models, the size of vascular defects is at least ~150 nm, compared to the ~5–9 nm pore size seen in metastases in the brain [83]. As a result, permeability values (rPSmax) for primary gliomas are approximately ten times higher than those for metastatic lesions, as shown in CT perfusion studies involving astrocytomas, GBMs, and metastases from lung, breast, and melanoma tumors [91,92]. Interestingly, astrocytoma values are closer to those of metastatic lesions than GBM.

### 3.9. Blood–Tumor Barrier in Brain Metastasis

Lung cancer, breast cancer, and melanoma are the most common cancers to metastasize to the brain, collectively accounting for the majority of brain metastasis cases, approximately 67–80% [93]. During the formation of brain metastases, systemic factors secreted by the primary tumor (PT) or early disseminated tumor cells play a critical role in altering the brain microenvironment [94]. These factors recruit immune cells, activate resident astrocytes and microglia, and remodel the ECM, thereby establishing a premetastatic niche conducive to tumor cell growth and survival [94]. For example, lipocalin-2 (LCN2) signaling from the PT triggers neuroinflammation in the brain metastatic niche by activating astrocytes. This process promotes the recruitment of LCN2-producing granulocytes from the bone marrow to the brain, enhancing the development of brain metastases. The BBB, while not entirely impenetrable, presents a significant barrier to invading metastatic cells. Studies show that the process of extravasation into the brain parenchyma beyond the endothelial layer takes notably longer than in other organs, ranging from 2 to 14 days depending on the primary tumor type [95,96]. Furthermore, although blood samples from patients show a high number of circulating tumor cells, only a small fraction of these cells successfully cross the BBB and establish metastatic lesions in the brain [97]. This discrepancy highlights the challenge tumor cells face in overcoming the BBB and surviving within the unique environment of the brain.

Circulating cancer cells adhere to the brain’s blood vessel endothelium and subsequently infiltrate the brain parenchyma. The transendothelial migration of tumor cells is thought to resemble the process by which leukocytes migrate through the endothelium, involving steps such as tethering, rolling, adhesion, and diapedesis [98]. However, the exact molecular mechanisms that facilitate tumor cell passage through this natural barrier remain poorly understood.

A recent study found that the enzyme ectonucleotide pyrophosphatase/phosphodiesterase 1 (ENPP1) secreted by a human epidermal growth factor receptor 2-positive (HER2+) breast cancer increased BBB permeability to facilitate brain colonization [99]. Similarly, brain metastatic cancer-cell-derived extracellular vesicles were shown to trigger BBB destruction, promoting the extravasation of cancer cells [100]. Once metastatic cells penetrate the brain parenchyma, tumor-induced angiogenesis leads to the development of immature blood vessels with increased fenestrations and reduced endothelial TJs, which together enhance vascular permeability [101]. Furthermore, the expression of efflux transporters is altered. A quantitative proteomic study of human brain metastases from lung and breast cancer patients found that P-gp/ABCB1 and BCRP/ABCG2 were undetectable in the microvessels of over 80% of samples when compared to the noncancerous cerebral cortex [102].

However, while the BTB in brain metastases is often described as “leaky,” it remains insufficiently permeable to permit the accumulation of therapeutically effective drug concentrations [83]. In fact, the brain concentrations achieved by most drugs used to treat brain metastasis (vemurafenib, paclitaxel (PTX), doxorubicin (DOX), palbociclib, cobimetinib, trametinib, dabrafenib) are less than 10% of their plasma concentrations [83]. Moreover, metastatic lesions exhibit substantial intra- and intertumoral heterogeneity within the same brain. Unlike primary tumors, where larger lesions typically cause more significant BBB disruption, there is no consistent correlation between the size of metastases and their degree of vascular permeability [103]. Generally, the vascular permeability in brain metastases is in the range of 1.1- to 100-fold, depending on the polarity and size of the marker [83]. Preclinical studies showed that cytotoxic levels of PTX, a highly lipophilic drug with a molecular mass of approximately 853 Da, were achieved only in a small subset (<10%) of the most permeable brain metastases, with drug concentrations exceeding 1000 ng/g [101]. Even in the most “leaky” brain metastases (~33-fold increased permeability), the drug’s penetration remained below 12% of that in a peripheral breast tumor [103]. Similarly, vinorelbine, also of lipophilic character and a molecular mass of 778 Da, showed limited efficacy, with brain metastasis exposure averaging four times that of normal brain tissue, yet reaching only about 8% of the exposure seen in non-barrier systemic metastases [104]. Water-soluble molecules, such as large antibodies (~150 kDa), face even greater permeability challenges when attempting to cross metastatic BTB defects. For instance, tissue distribution studies with ^125^I-trastuzumab showed that only about 3% of the injected dose reached normal brain tissue, and even in brain tumors, uptake was limited to approximately 5% of the dose [105]. Even when therapeutic agents penetrate the brain, their distribution within metastatic lesions is markedly heterogeneous. Morikawa et al. reported up to a 60-fold difference in lapatinib concentrations (1–63 μM) across resected brain metastases from just four patients following oral administration [106]. Similarly, carbon-11-labeled lapatinib positron emission tomography (PET) imaging revealed a seven-fold variability (0.5–3.4 μM) in intratumoral uptake [107].

### 3.10. Tumor Microenvironment

Beyond the restrictive nature of the BBB and BTB, TME poses formidable physical and chemical barriers to drug delivery. The absence of effective lymphatic clearance prevents fluid drainage, leading to fluid retention and elevated interstitial fluid pressure (IFP), which creates a physical barrier to drug penetration [108]. Additionally, the leaky and abnormal tumor vasculature allows plasma proteins and fluid to escape into the surrounding tissue, further increasing IFP and impairing blood flow, which complicates the delivery of therapeutic agents. High IFP reduces the transvascular transport of therapeutic agents, leading to decreased drug accumulation within the tumor. Moreover, the density and stiffness of the ECM constitute an additional barrier that restricts drug diffusion, reducing the effectiveness of chemotherapy and other therapeutic approaches [109]. These aspects collectively reduce the driving force for fluids and solutes to extravasate from the vasculature into brain tissues, posing a formidable barrier to effective drug delivery and the accumulation of therapeutic agents, ultimately contributing to treatment resistance. Overcoming these physical barriers is essential to enabling successful delivery and accumulation of therapeutic agents at the diseased site.

The high metabolic demands of brain tumors result in the acidification of the TME, forming hypoxic zones often surrounded by hypercellular rings of actively migrating tumor cells [110,111,112]. In gliomas, inadequate vascularization contributes to the hypoxic state, but even in the presence of sufficient oxygen, tumor cells preferentially undergo glycolysis over oxidative phosphorylation—a phenomenon known as the Warburg effect—which leads to lactate accumulation and further acidification of the extracellular milieu [110]. Notably, this pathophysiological pattern is not unique to primary brain tumors: recent evidence has shown that brain metastases also exhibit pronounced hypoxia and oxidative stress, as demonstrated by PET imaging with [64Cu][Cu(ATSM)] and proteomic analyses in a lung-derived brain metastasis model [113].

The consequences of hypoxic conditions are two-fold. Firstly, in GBM, hypoxia drives the formation of pseudopalisading tumor cells—radially aligned, highly migratory cells that cluster around necrotic cores [111]. These cells are pro-angiogenic, therapy-resistant, and contribute to tumor progression, invasion, and treatment failure [112]. In contrast, although pseudopalisading structures are not a feature of brain metastases, hypoxia in metastatic lesions similarly promotes angiogenesis, immune evasion, and cellular adaptation to low oxygen environments. Hypoxia-inducible factors (HIFs) in metastatic brain tumors regulate pathways that enhance tumor cell survival, vascular remodeling, and resistance to radiation and chemotherapy [114]. Due to poor perfusion, these hypoxic areas—whether in primary or secondary brain tumors—are often poorly accessible to circulating drugs, limiting therapeutic efficacy. Secondly, even if some amount of the circulating drug manages to reach these areas, the acidic microenvironment significantly affects drug bioavailability due to the “ion trapping” phenomenon [115,116]. In acidic surroundings, weak base drugs become protonated and thus charged. Given that charged forms of ionizable drugs are less effective at crossing the plasma membrane of cells, these drugs accumulate outside the tumor cells, reducing their effectiveness within the tumor [115,116]. Thus, the tumor’s acidity can act as a barrier to weak base drugs, such as anthracyclines and vinca alkaloids, while favoring weak acid drugs like chlorambucil [117,118]. Beyond its impact on small-molecule drugs, an acidic TME can also significantly influence the behavior of nanoparticles (NPs) [119]. Acidic pH can destabilize certain NPs, leading to premature drug release or uncontrolled aggregation within the tumor interstitium [119]. Such aggregation can hinder deep tissue penetration and limit uniform therapeutic distribution, as NPs become trapped near blood vessels (“perivascular accumulation”) and fail to reach hypoxic or deeply infiltrated tumor regions that harbor the most aggressive and therapy-resistant tumor cells. While pH-responsive NPs can be specifically engineered to exploit the acidic environment for controlled drug release, unintended destabilization remains a risk, potentially compromising therapeutic efficacy. Consequently, the design of nanocarriers intended for brain tumor delivery must address not only BBB traversal but also stability and functional performance within the hostile acidic milieu of the TME. These caveats highlight that effective treatment strategies must overcome not only the vascular barriers but also the hostile biochemical and biophysical conditions within the TME to achieve sufficient intratumoral drug retention and therapeutic activity.

### 3.11. Methods to Enhance Drug Delivery in Brain Tumors

**Figure 4:**
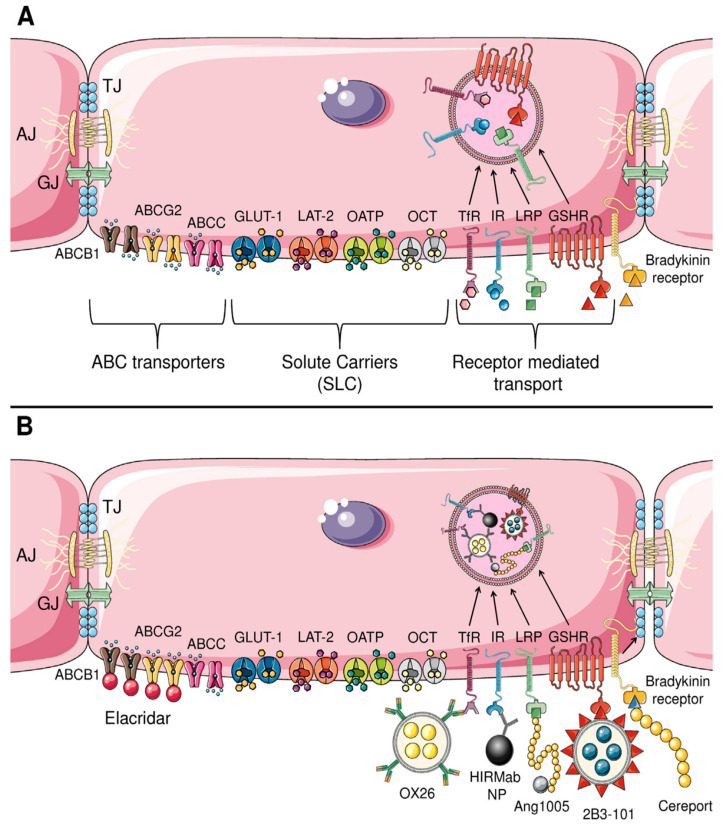
Exploiting endogenous BBB mechanisms for enhanced brain drug delivery (adapted from van Tellingen et al. [23]). (**A**) The BBB restricts the entry of exogenous compounds mainly via ABC efflux transporters on the luminal surface of bECs. These cells also express diverse solute carrier (SLC) influx transporters responsible for the uptake of essential nutrients, including glucose (GLUT1), amino acids (LAT2), organic anions (OATP family), and organic cations (OCT family). Additionally, receptor-mediated transcytosis pathways facilitate the transport of macromolecules through receptors such as the transferrin receptor (TfR), insulin receptor (IR), low-density lipoprotein receptor-related protein (LRP), and glutathione receptor (GSHR). (**B**) Therapeutic strategies have been developed to exploit or bypass these mechanisms for improved brain drug delivery. Elacridar inhibits the ABCB1 and ABCG2 efflux pumps, enhancing the brain uptake of small molecules. Receptor-mediated transcytosis is harnessed by agents such as OX26 (anti-TfR antibody), human insulin receptor monoclonal antibody-conjugated nanoparticles (HIRMab NPs), Ang1005, and 2B3-101. Cereport targets bradykinin receptor signaling to transiently loosen tight junctions, increasing paracellular permeability.

Although the restrictive nature of the BBB and BTB has been recognized for over a century, the development of effective drug delivery strategies targeting these barriers has historically lagged behind other areas of oncology. This delay was largely due to technical limitations and an incomplete understanding of the structural and functional complexity of these interfaces. In recent decades, however, significant advances have deepened our knowledge of BBB and BTB biology, including the roles of tight junctions, efflux transporters, and regulated transcytosis. Notably, RMT, CMT, and AMT transport mechanisms have emerged as promising targets for improving CNS drug penetration. These insights have spurred a wide range of experimental and translational approaches aimed at enhancing drug delivery to the brain (Figure 4). While these innovations have yet to produce a definitive breakthrough in brain tumor therapy, the field remains dynamic, with considerable potential to transform therapeutic outcomes in neuro-oncology. The following sections examine these pharmacological strategies in detail.

### 3.12. Bradykinin Receptor

Bradykinin (BK) activates B2 receptors located on the abluminal side of the BBB, triggering a cascade of events, including the release of nitric oxide (NO) and other vasoactive mediators [120,121,122]. This activation leads to the disruption of TJs between bECs, resulting in increased BBB permeability [120]. The topical application of BK to the pial surface of the brain was found to induce BBB disruption, allowing the passage of small molecules like fluorescein while restricting larger macromolecules such as albumin [120]. Preclinical studies have shown that intracarotid BK administration preferentially opens the BBB in tumor tissue compared to the normal brain [121,122]. This selective effect occurs because tumor vessels are more permeable, allowing BK to reach and activate B2 receptors on the abluminal side of bECs [12]. In healthy brain tissue, however, the absence of B2 receptors on the luminal side of endothelial cells prevents BK from having the same disruptive effect on the BBB. Given its rapid degradation and a plasma half-life of approximately 15 s, a more stable and potent analog, RMP-7 (Cereport/lobradimil), has been developed [123]. Intravenous or intracarotid RMP-7 has been shown to increase the delivery of carboplatin in experimental brain tumors [124]. In patients with recurrent malignant gliomas, intracarotid infusion of RMP-7 led to a 46 ± 42% (mean ± standard deviation) increase in the delivery of [68Ga]-EDTA, a permeability marker, as measured by PET [125]. Given its physicochemical similarity to carboplatin—including molecular size, charge, and solubility—this study provided the rationale for subsequent clinical trials investigating RMP-7-facilitated carboplatin delivery in adults with gliomas [126,127], and in children with brainstem gliomas in combination with radiotherapy [128]. Yet, clinical efficacy has not been demonstrated. In a randomized, double-blind, placebo-controlled Phase II trial, 122 adults with recurrent high grade gliomas (HGGs) treated with carboplatin plus RMP-7 (300 ng/kg) had a median time to progression of 9.7 weeks compared to 8.0 weeks in the carboplatin plus placebo group [129]. Given that a 300 ng/kg dose of RMP-7 failed to enhance the efficacy of carboplatin, it was hypothesized that higher doses might be required to achieve meaningful drug penetration. However, a subsequent single-arm Phase II trial involving 38 children with high-grade and brainstem gliomas—the last clinical study to evaluate RMP-7—demonstrated no antitumor activity when RMP-7 was combined with carboplatin at a dose of 600 ng/kg [130]. The lack of clinical benefit remains unclear, with possible explanations including drug-scheduling challenges, as the window of BTB/BBB opening induced by RMP7 is relatively short, and a potential insufficient increase in drug delivery despite barrier disruption. As no further studies have been conducted, the efficacy of BK analogs in enhancing drug delivery to brain tumors remains uncertain.

Recently, a novel BK analog (NG291), exhibiting a high affinity for the B2 receptor, has been shown to preclinically incite BBB disruption without contributing to the risks associated with BBB disruption via BK receptor agonism, such as vasogenic edema and neuroinflammation [131]. Further studies are essential to evaluate this novel agent. Targeting the B2 receptor may offer potential benefits for patients with pre-existing BBB disruption, as it can enhance drug delivery and reduce tumor size. However, this approach will not be effective for improving drug concentrations in tumor regions protected by an intact BBB, as the B2 receptors remain inaccessible in these areas. A major concern surrounding the use of BK analogs to disrupt the BBB is their potential to promote the invasive migration of glioma cells through B2 receptor activation [132,133,134]. BK has been shown to stimulate glioma cell migration both in vitro and in acute brain slices, facilitating the perivascular migration of invading tumor cells—a key mechanism in glioma cell dispersion [132,133,134]. Consequently, BK analogs could inadvertently exacerbate tumor invasiveness, making glioma cells more prone to infiltration.

### 3.13. Adenosine Receptor

Observations that extracellular adenosine facilitates lymphocyte entry into the CNS in experimental autoimmune encephalomyelitis prompted the hypothesis that targeting adenosine receptors (ARs)—expressed on both the luminal and abluminal surfaces of the BBB—could be leveraged to transiently enhance BBB permeability [135,136]. Among the four G-protein-coupled AR subtypes, A1 and A2A play key roles in modulating barrier function through opposing effects on downstream signaling pathways [137]. The A2A receptor, in particular, is highly expressed and functionally active in both the brain and heart, where it serves as a critical regulator of local vasodilation [137]. Activation of A2A receptors has been shown to increase BBB permeability through several mechanisms: modulating TJ integrity, inducing cytoskeletal rearrangements, and promoting nitric-oxide-mediated vasodilation. These effects are largely mediated through cAMP-dependent signaling, leading to increased paracellular permeability and facilitating the transendothelial transport of immune cells and therapeutic agents [136,138]. The A2A adenosine receptor agonist regadenoson (Lexiscan), administered at a dose of 0.0005 mg/kg, has been shown to enhance BBB permeability—permitting the passage of 70 kDa dextran in murine and rat models [136], facilitating T cell transmigration across a human in vitro BBB model [137], and increasing brain concentrations of TMZ by 60% in non-tumor-bearing rats [139]. This preclinical evidence prompted early clinical investigations of regadenoson to enhance CNS drug delivery. However, imaging studies using SPECT and CT tracers that do not cross an intact BBB showed no detectable increase in CNS permeability following systemic regadenoson administration [140]. Similarly, in patients with recurrent HGGs undergoing surgical resection, regadenoson co-administered with oral TMZ failed to increase intraparenchymal drug concentrations, as assessed by peritumoral microdialysis [141]. The discrepancy between preclinical and clinical studies can be attributed to interspecies variability in BBB structure and function—particularly in the expression, localization, and responsiveness of CNS A2A receptors. Moreover, the dose of regadenoson used in these clinical studies corresponds to the FDA-approved dosage for cardiac stress testing (0.4 mg). The optimal dose of regadenoson required to modulate the integrity of the BBB in humans remains undefined. A preclinical study has shown a non-linear, dose-dependent response, suggesting the presence of a narrow “sweet spot” in which BBB permeability is transiently enhanced. Both lower and higher doses outside this range appear less effective, pointing to a complex pharmacodynamic relationship that varies across species [136]. To define the optimal dosing strategy for transient BBB modulation in humans, a Phase I clinical trial (NCT03971734) investigated escalating doses of regadenoson (0.05–1.4 mg) in patients with HGGs [137]. Regadenoson was well tolerated at all administered doses. The primary endpoint—a 10-fold increase in vascular permeability, assessed by the forward volume transfer constant (K^trans) using dynamic contrast-enhanced MRI (DCE-MRI)—was not achieved. A secondary endpoint, defined as a greater than 100% increase in Z-score-normalized signal intensity on T1-weighted subtraction maps, was also unmet; the observed mean increase was 75% ± 22% (*p* = 0.016). Although these findings suggest a modest pharmacodynamic effect, the extent of BBB disruption was considered insufficient to meaningfully improve CNS drug delivery [137]. Future investigations should explore the potential of repeated regadenoson administration and assess the efficacy of combination strategies involving adenosine and bradykinin analogs to achieve more effective BBB modulation.

### 3.14. Sphingosine-1-Phosphate Receptor

Sphingosine-1-phosphate (S1P) is a small phospholipid signaling molecule that interacts with five G-protein-coupled receptors (GPCRs) to produce various cell-type-specific effects [142,143]. Human bECs express S1P receptors 1 (S1P1), 2 (S1P2), 4 (S1P4), and 5 (S1P5), while S1P3 is predominantly expressed by pericytes and VSMCs [142,143,144]. Among these, S1P1 is the most widely expressed receptor and has been extensively studied for its role in modulating BBB integrity and permeability [144]. Research on cultured bECs suggests that S1P1 regulates AJs by controlling the distribution of VE-cadherin [144].

A study demonstrated that the knockout of endothelial S1P1 in mice led to a breach of the BBB for low-molecular-weight fluorescent tracers (<3 kDa), while larger tracers (>10 kDa) were still excluded [142]. These effects were similar to those observed in double knockdowns of Cldn5 and occludin [145]. While the cytoskeletal modifications of TJ proteins are believed to contribute to these changes, the exact mechanisms by which S1P1 knockout alters TJ protein localization in bECs remain unclear [142]. Importantly, the study did not observe signs of inflammation or gliosis in the mouse brains, suggesting that targeting S1P1 could selectively and reversibly open the BBB to small molecules without allowing larger, potentially harmful macromolecules into the brain parenchyma. In fact, the same study found that fingolimod, a functional antagonist of S1P1, could facilitate selective and reversible BBB opening for small molecules [142]. These findings highlight the therapeutic potential of modulating S1P receptor signaling—particularly S1P1—as a means to achieve controlled, size-selective BBB permeability, warranting further investigation into the underlying molecular mechanisms and translational applications.

### 3.15. Inhibition of Drug Efflux Transporters

The BBB expresses a range of efflux transporters that collectively restrict the entry of xenobiotics and therapeutic agents into the CNS. Among these, the ABC transporter family plays a principal role, with P-gp/ABCB1 being the most abundant and functionally dominant efflux pump at the BBB [11,28]. Chemotherapeutic agents such as DOX, PTX, and vinblastine can induce P-gp/ABCB1 overexpression, contributing to the development of multidrug resistance (MDR) [145,146]. Alongside P-gp/ABCB1, other ABC transporters—including BCRP/ABCG2 and various multidrug-resistance-associated proteins (MRPs)—further reduce drug accumulation in the CNS, collectively diminishing the efficacy of many anticancer agents. Various chemotherapy drugs are substrates for these transporters. For example, vincristine, DOX, and etoposide are substrates for P-gp/ABCB1 [147], while methotrexate is a substrate for MRP4 [148], and imatinib, gefitinib, and nilotinib are substrates for BCRP/ABCG2 [149,150]. Notably, TMZ is a substrate for both P-gp/ABCB1 and BCRP/ABCG2 [151].

As a result, pharmacological inhibition of these transporters has been proposed as a strategy to enhance drug retention in the brain by limiting their active efflux back into the circulation [11,147]. Valspodar, a first-generation P-gp/ABCB1 inhibitor, demonstrated in preclinical models an up to eight-fold increase in PTX brain concentrations. However, it also significantly increased drug exposure in peripheral tissues such as the liver, kidneys, and plasma, raising concerns over systemic toxicity [152]. Additionally, valspodar interferes with cytochrome P450 enzymes, impairing drug clearance and necessitating dose reductions in co-administered cytotoxic agents.

To overcome these limitations, third-generation ABCB1 inhibitors—tariquidar, elacridar, and zosuquidar—have been developed to selectively target P-gp/ABCB1 at the BBB while minimizing off-target interactions, particularly with cytochrome P450 enzymes [153,154,155]. These non-substrate inhibitors bind to distinct allosteric sites on P-gp/ABCB1, inducing conformational changes that impair transporter function. Among them, elacridar is the most potent, achieving complete P-gp/ABCB1 inhibition at plasma concentrations around 1.0 μM, compared to over 4.0 μM for tariquidar. Elacridar also inhibits BCRP/ABCG2, albeit with lower potency, making it more effective for enhancing the brain penetration of P-gp/ABCB1 substrates than those primarily effluxed by BCRP/ABCG2. In contrast, zosuquidar weakly inhibits organic cation transporters (OCT1–3) [154], while tariquidar is itself a substrate for ABCG2, which limits its effectiveness as a brain delivery enhancer [155].

Despite promising results in animal models—demonstrating increased brain accumulation of PTX [156,157], antiepileptics [158], virostatics [159], dasatinib [160], sorafenib [161], sunitinib [162], TMZ [163], olaparib [164], and topotecan [165]—these studies often relied on supratherapeutic doses that induced functional P-gp/ABCB1 knockout. This exaggerates the translational potential of such inhibitors in humans [166]. Supporting this, PET imaging studies revealed species-dependent differences in BBB P-gp/ABCB1 function, with significantly greater increases in (R)-[11C]verapamil brain distribution observed in rats compared to humans at equivalent plasma levels of tariquidar [167].

Clinical investigations into third-generation P-gp/ABCB1 inhibitors remain limited [168,169]. In one study of healthy volunteers, a 2 mg/kg dose of tariquidar increased (R)-[11C]verapamil brain penetration by approximately 25%, while higher doses of 4 and 6 mg/kg resulted in 2- and 4-fold increases, respectively [168].

A key prerequisite for the success of efflux inhibition strategies is the complete blockade of all relevant transporters for a given drug. Members of the MRP family, including MRP1 and MRP4, can further restrict CNS drug penetration, complicating the pharmacological modulation of efflux. Therefore, for any agent intended to target the brain, it is imperative to identify all active efflux pathways and tailor inhibition strategies accordingly. Only by ensuring full suppression of these systems can meaningful improvements in brain drug delivery be achieved.

While ABC transporter inhibitors show considerable promise in preclinical studies, their clinical translation has been hindered by their limited bioavailability, incomplete inhibition of efflux activity, and lack of efficacy across the entire spectrum of efflux transporters at the BBB. These challenges underscore the need for alternative or adjunctive strategies that modulate barrier permeability through mechanistically distinct pathways. Tesmilifene, a tamoxifen analog and histamine antagonist with cytotoxic properties, has been shown to increase BBB and BTB permeability [170,171]. In a rat RG2 glioma model, tesmilifene reduced transendothelial electrical resistance and inhibited key efflux transporters—P-gp/ABCB1, BCRP/ABCG2, and MRP1—resulting in a 2.7-fold increase in fluorescein accumulation and enhanced albumin permeability within tumor tissue [172]. It also downregulated the mRNA expression of tight junction proteins, solute carriers, and metabolic enzymes critical to BBB integrity. Although its exact mechanism remains unclear, preliminary evidence implicates the MAPK/ERK and PI3K/AKT pathways [172]. Despite these promising results, no further preclinical studies have explored tesmilifene’s potential to enhance CNS drug delivery, highlighting the need for continued investigation.

### 3.16. Nanotechnology for Brain Tumor Drug Delivery: Passive and Active Targeting Strategies

Significant advancements in polymer science and nanotechnology over the past few decades have propelled NPs to the forefront of drug delivery research [115,173]. Their unique properties—such as their nanoscale size, biocompatibility, and low toxicity—make them particularly well-suited to enhancing drug delivery to the brain [115,173]. Notably, NPs can extend the half-life of therapeutic agents and enable controlled, sustained release, offering significant advantages for drug delivery. Moreover, NPs can be engineered to exploit the acidic TME by releasing their payload preferentially at low pH, converting surface charge to enhance uptake, or undergoing structural transformations that improve penetration and intratumoral accumulation—thus turning a pathological feature into a delivery advantage. One promising strategy to improve the pharmacokinetics of NPs is surface hydration, or steric modification [115]. This involves the addition of neutral or hydrophilic polymers to the NP surface (either adsorbed or covalently linked), creating an extra hydration layer that shields NPs from plasma opsonins. This modification reduces the uptake of NPs by the RES, thereby extending their circulation time in the plasma [115]. Fang et al. demonstrated this effect by encapsulating TMZ in chitosan-based NPs, which increased the drug’s plasma half-life from 1.8 h (with free TMZ) to 13.4 h [174]. This technique can be applied to a broad range of hydrophilic and hydrophobic drugs, including immunotherapies, using various methods such as encapsulation, covalent attachment, or surface adsorption. Lastly, NPs can be formulated with or functionalized by natural bioactive molecules—such as alkaloids, flavonoids, coumarins, resins, saponins, and terpenoids—or by polymers like PEG, which have been shown to inhibit P-gp/ABCB1 activity [28]. An alternative strategy involves encapsulating therapeutic agents together with known P-gp/ABCB1 inhibitors within the NP core, thereby enhancing intracellular drug accumulation and overcoming efflux-mediated resistance at the BBB.

Nanotechnology offers a diverse array of carriers, including dendrimers, micelles, carbon nanotubes, exosomes, and liposomes, all of which show promise for enhancing BBB/BTB penetration and delivering diverse therapeutic agents [11,173,174]. These carriers can be designed to exploit two primary strategies for drug delivery to the CNS: passive and active targeting [11].

### 3.17. Passive Targeting

Passive targeting involves designing NPs that are small (10–100 nm) and lipophilic enough to cross the BBB by passive diffusion. However, it has become evident that simply reducing NP size or increasing lipophilicity is insufficient to achieve effective BBB penetration [115,175,176]. The enhancement of permeability and retention (EPR) effect has similarly shown limited success in improving the accumulation of NPs within brain tumors [175]. The EPR effect is based on the idea that NPs of a certain size preferentially accumulate in tumor tissue [175]. There are two prerequisites for EPR: (i) abnormal leaky tumor vessels and (ii) improper lymphatic drainage. Theoretically, this would lead to the extravasation of NPs and reduced lymphatic clearance. However, decades of research have demonstrated that the EPR effect is highly heterogeneous, both within individual tumors and across different types of tumors [175]. Moreover, its clinical relevance has been overestimated, as it is much more significant in experimental small animal tumor models than in human tumors [175]. Key reasons for this discrepancy include differences between preclinical animal models and human tumors, such as the variability in the presence of fenestrations in the tumor endothelium, hypoxic and acidic TMEs, heterogeneous pericyte and BM coverage, and elevated IFP from dense extracellular matrices [115,175,176,177]. While the EPR effect can be effective in some peripheral solid tumors, its relevance in brain tumors is significantly limited due to several factors. First, only a subset of the tumor-associated vasculature within brain tumors exhibits the fenestrations necessary for NP extravasation, resulting in heterogeneous and often inadequate permeability across the BTB [7]. Additionally, brain tumors exhibit elevated IFP, which counteracts the passive diffusion of NPs, further limiting their accumulation via the EPR effect [108]. Finally, unlike peripheral tumors, which benefit from poor lymphatic drainage to retain macromolecules, the brain lacks a conventional lymphatic system [178,179]. Instead, NPs that do penetrate may be rapidly cleared through CSF pathways, reducing their retention within the tumor [178,179]. These physiological factors collectively undermine the effectiveness of the EPR effect in brain tumors, necessitating alternative strategies for NP-based drug delivery. Consequently, passive targeting strategies have been largely abandoned in favor of active targeting strategies, which are now at the forefront of neuro-oncology [11,12].

### 3.18. Active Targeting

Active targeting strategies enhance the specificity and efficiency of drug delivery to the brain by leveraging endogenous transport mechanisms within the BBB [115]. This approach typically involves conjugating therapeutic agents or NPs to specific ligands (e.g., antibodies, peptides, or small molecules) that bind to receptors or transporters abundantly expressed on the surface of bECs. Upon binding, these conjugates are internalized and trafficked across the BBB, effectively “tricking” the barrier into transporting the drug into the brain—hence the name “Trojan Horse strategy” [11]. While RMT is the most widely studied, other transport systems, such as CMT—utilizing solute carriers such as glucose transporter 1 (GLUT1) to shuttle drugs across the BBB—or AMT—exploiting electrostatic interactions for uptake—also represent viable pathways for drug delivery [11]. Advances in nanotechnology and antibody engineering have expanded the arsenal of targeted drug delivery systems, enabling the development of NP– or drug–ligand conjugates tailored to target specific transport mechanisms. These approaches facilitate drug penetration in a controlled manner while preserving BBB integrity. The next section will explore these mechanisms in detail, evaluating their advantages and limitations in brain tumor therapy.

### 3.19. Carrier-Mediated Transport

CMT exploits the high affinity of low-molecular-weight nutrients and molecules for specialized transporters at the BBB [11]. These transporters belong to the solute carrier (SLC) transporters superfamily, the second-largest membrane protein family after G-protein-coupled receptors, comprising over 400 genes across 60 families [11]. Despite the remarkable diversity of the SLC superfamily, some transporters exhibit overlapping functions [11,180]. The GLUT family alone comprises more than ten glucose transporters, highlighting the complexity of nutrient transport across the BBB. Given this intricacy, effective drug delivery via CMT requires careful validation to ensure that the substrate transporter profile (STP) observed in vivo aligns with in vitro models expressing the SLC transporter of interest [11]. Several carriers have been identified as promising targets, including GLUT1, large neutral amino acid transporter (LAT1), monocarboxylate transporter (MCT1), purine nucleoside transporter (CNT2), choline transporter (CTL1), and various SLC transporters involved in vitamin transport [11]. While other SLC transporters are emerging as potential targets, GLUT1 and LAT1 remain at the forefront due to their well-characterized mechanisms and demonstrated efficacy in preclinical and clinical studies.

### 3.20. Glucose Transporter 1

Encoded by *SLC2A1*, GLUT1 is a high-affinity glucose transporter that has emerged as a promising target for CMT due to its significantly higher expression at the BBB compared to many other receptors and transporters—approximately 100-fold greater than the transferrin receptor (TfR) [181,182]. GLUT1 is abundantly expressed on both the luminal and abluminal membranes of bECs and in the astrocytic end-feet, facilitating >95% of glucose transport across the BBB [11]. Moreover, glioma cells exhibit a significantly elevated expression of GLUT, particularly in the necrotic and hypoxic regions [183]. Drugs designed to utilize GLUT1 for BBB penetration typically rely on molecular conjugation to glucose or structural mimicry [11]. The latter approach remains largely unexplored for brain tumors due to the challenge of balancing BBB permeability with therapeutic efficacy. Conjugation is preferred as it offers greater control over drug pharmacokinetics and bioavailability. However, GLUT1’s narrow pore dimensions (0.8 × 1.5 nm) impose a size constraint, allowing only small molecules such as D-glucose (~1 nm long) to be transported [11,184]. Consequently, GLUT1-targeting nanocarriers exceed the transporter’s size limit, explaining the minimal brain penetration reported in studies (~0.4% dose/g-brain), with no significant improvements reported to date [185,186].

Anraku et al. devised a strategy to enhance brain uptake of glucose-functionalized NPs by leveraging three key principles: (1) multivalent glucose conjugation to the nanocarrier surface to strengthen adherence to bECs via GLUT1 interactions, with 25%Gluc(6)/m identified as the optimal glucose surface density; (2) administration under hypoglycemic conditions, which induces a >50% increase in luminal GLUT1 expression on the BBB; (3) subsequent glucose elevation to reduce the concentration gradient that would otherwise drive glucose-conjugated NPs back into circulation, thereby enhancing cerebral retention [187]. In murine models, authors reported a brain accumulation rate of 6% dose/g-brain, a 56-fold increase compared to free-feeding controls [188]. This surpasses previously reported uptake rates for both glucose-based (~0.4% dose/g-brain) [185] and peptide–ligand-based (~1.0% dose/g-brain) systems [189,190]. The same strategy has been successfully applied for antisense oligonucleotide (ASO) delivery, demonstrating robust accumulation in the cerebral cortex and hippocampus following intravenous administration [191]. Interestingly, rather than direct GLUT1-mediated translocation across the BBB, these NPs underwent partial transcytosis via GLUT1 recycling, a process triggered by glycemic modulation and involving clathrin-independent endocytosis [188]. This behavior is characteristic of RMT rather than classical CMT, reinforcing the notion that CMT alone may be insufficient for achieving therapeutic brain drug levels [11].

Further studies are warranted to refine this strategy in disease-relevant models, as GLUT1 expression varies considerably between normal and pathological states. While gliomas exhibit GLUT1 upregulation, GBM patients frequently experience hyperglycemia due to high-dose glucocorticoid therapy for peritumoral edema [192]. These metabolic alterations could impact transporter availability and necessitate additional pharmacokinetic optimization for clinical translation.

### 3.21. L-Type Amino Acid Transporter 1

LAT1 is abundantly expressed on both the luminal and abluminal membranes of the BBB, where it exhibits a higher affinity for its substrates than in peripheral tissues [193]. Among BBB amino acid influx transporters, LAT1 has the highest transport capacity [194]. LAT1 overexpression has been widely reported in gliomas, with quantitative PCR analyses revealing a 40- to 400-fold increase in LAT1 expression in GBM compared to normal brain tissue, correlating with tumor grade [195]. Beyond serving as a nutrient transporter, LAT1 promotes tumor growth, migration, and angiogenesis.

**Figure 5:**
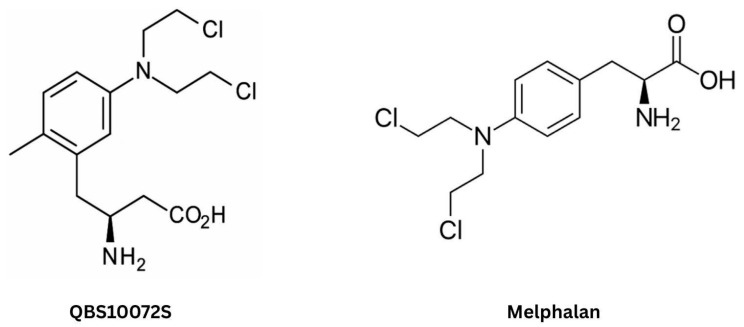
The molecular structure of QBS10072S and melphalan.

The dual expression of LAT1 in the BBB and glioma cells makes it a promising target for drug delivery. Several LAT1 substrates—such as gabapentin, BCH (2-amino-2-norbornanecarboxylic acid), and levodopa—cross the BBB at pharmacologically significant levels [11]. Among chemotherapeutic agents, melphalan is the only alkylating drug reported to utilize LAT1, showing some efficacy in experimental brain cancer models [196,197,198] but ultimately failing in GBM clinical trials [199]. This failure is attributed to its poor LAT1 affinity, largely due to the para-substitution of its N-(bis-(2-chloroethyl)amino) group on the phenyl ring, which limits BBB penetration and reduces drug accumulation in the peritumoral environment [200]. Additionally, melphalan lacks LAT1 selectivity, potentially entering healthy brain cells via alternative transporters like LAT2, increasing the risk of neurotoxicity and limiting tolerability [200]. To overcome these limitations, QBS10072S was designed to retain the cytotoxic moiety of melphalan while enhancing LAT1-mediated transport (Figure 5). This novel compound exhibits high BBB permeability, preferentially accumulates in orthotopic GBM models with minimal off-target toxicity, and circumvents O6-methylguanine-DNA methyltransferase (MGMT)-mediated resistance through dual mechanisms of DNA crosslinking and alkylation [200]. In preclinical studies, QBS10072S significantly inhibited tumor growth and prolonged survival, including in a leptomeningeal disease model where it was effective despite an intact BBB. As of now, QBS10072S is under investigation in a Phase I clinical trial involving previously treated patients with advanced or metastatic cancers characterized by high LAT1 expression, as well as those with relapsed or refractory GBM (ClinicalTrials.gov Identifier: NCT04430842). In parallel, the INSIGhT trial (NCT02977780) recently reported that QBS10072S was well tolerated in patients with newly diagnosed, unmethylated MGMT GBM. Based on these findings, a recommended Phase II dose (RP2D) of 18 mg/m^2^ was established and is now being evaluated in an ongoing Phase II treatment arm [201].

LAT1 has also been explored as a target for NP-based drug delivery [202,203,204,205,206,207]. In a GBM model, LAT1-targeted NPs encapsulating TMZ and sorafenib achieved pharmacologically active drug levels beyond the BBB, inducing a synergistic antitumor effect [206]. Similarly, LAT1-mediated delivery of ASO-loaded NPs resulted in a ~64-fold increase in brain uptake compared to ASOs administered without an NP carrier. These findings underscore LAT1’s potential to enhance NP accumulation in the CNS [207].

Since LAT1 may facilitate intracellular NP uptake through CMT or endocytosis—similar to GLUT1—further research is needed to elucidate the precise mechanisms governing LAT1-mediated transport. A deeper understanding of LAT1-substrate interactions could inform the rational design of NPs, optimizing drug delivery to the CNS.

Both compounds share a bifunctional tertiary bis(2-chloroethyl)amine alkylating group responsible for their cytotoxic effects. Melphalan features a phenylalanine-like amino acid backbone, while QBS10072S is chemically modified at the amino acid moiety to enhance recognition and transport by the LAT1. This modification enables QBS10072S to more effectively cross the blood–brain barrier and selectively target LAT1-expressing glioblastoma cells, while retaining the alkylating activity of melphalan.

### 3.22. Choline Transporter-like Protein 1

Choline is vital for CNS function, serving both as a precursor for acetylcholine and as a key component of membrane phospholipids. As a charged cation, it requires facilitated transport across the BBB via CMT. Studies employing the internal carotid artery perfusion method have confirmed active choline transport in rats, albeit with a low transport capacity (Vmax = 2.7 nmol/min/g) [11,208]. The choline transporter, particularly the CTL1, has been investigated as a potential target for improving drug delivery across the BBB. While CTL1 mRNA and protein have been detected in cultured human bECs [209], quantitative proteomics analyses suggest that its in vivo protein abundance at the BBB is very low, similar to other low-capacity transporters such as MCT1 and CNT2 [210]. Although definitive evidence for CTL1-mediated transport of choline or choline-like drugs across the BBB is lacking, it has been explored as a target for NP-based drug delivery for two reasons: (i) its expression in glioma cells [211]; (ii) the low physiological plasma concentration of choline (~25% of the Michaelis–Menten constant, Km), which leaves CTL1 relatively unoccupied, potentially enabling the facilitated transport of choline derivatives without significantly disrupting endogenous choline homeostasis [208].

A biodistribution study showed that choline derivative (CD)-conjugated polyethylene glycol (PEG) micelles carrying DOX resulted in a 24-fold higher concentration of DOX compared to the free drug [212]. This formulation also led to the most significant antitumor effects and the longest median survival in a mouse model of GBM (U87-luci). CD-PEG-DOX micelles delivered 2.37 times more DOX when compared to non-conjugated micelles, highlighting the advantage of using the targeting ligand for better drug delivery. Recently, Wu et al. developed NPs decorated with acetylcholine and choline analogs to simultaneously target CTL1 and nicotinic acetylcholine receptors (nAChRs), reporting a 42-fold increase in brain accumulation of bovine serum albumin and uniform distribution in the neuronal extracellular space 24 h post-administration [213]. Similarly, Wang et al. designed NPs incorporating the choline analog 2-methacryloyloxyethyl phosphorylcholine (MPC), conjugated to anti-programmed death-ligand 1 (anti-PD-L1) via a pH-sensitive traceless linker. These NPs exhibited enhanced BBB penetration in an orthotopic glioma model, effectively releasing anti-PD-L1 in the acidic TME [214]. While CTL1 can be considered a potential CMT target for glioma therapy, the lack of definitive evidence for abundant expression on bECs in vivo warrants further investigation before it can be validated as a candidate for improving the BBB penetration of therapeutic molecules.

### 3.23. Other Transporters

The Na^+^-dependent carnitine transporter novel organic cation transporter 2 (OCTN2) is expressed at the BBB, where it facilitates the uptake of the essential metabolite L-carnitine from systemic circulation into the brain, and it is also present in GBM cells [215,216]. L-carnitine-conjugated poly(lactic-co-glycolic acid) (PLGA) NPs loaded with PTX achieved up to a ∼13-fold increase in brain accumulation compared to free PTX and an ∼11-fold increase compared to non-targeted PLGA NPs in T98G GBM cells, significantly enhancing antitumor efficacy [217]. The substantial inhibition of NP uptake by chlorpromazine (a clathrin-mediated endocytosis inhibitor) and indomethacin (reported to inhibit caveolin-mediated pathways), along with the minimal effects of colchicine and quercetin (targeting macropinocytosis and nonspecific endocytic routes), suggests that RMT constitutes the primary mechanism of NP internalization, rather than CMT.

Glutathione (GSH), a natural antioxidant present at high concentrations in the brain, is unable to cross the BBB via passive diffusion due to its high hydrophilicity and negative charge at physiological pH [218,219]. Both Na^+^-dependent and Na^+^-independent transport mechanisms have been proposed to mediate GSH transport across the luminal membrane of bECs [218]. Additionally, MRPs, particularly MRP1, MRP2, and MRP4, are recognized as key contributors to GSH efflux at the BBB. Building on this understanding, GSH has been explored as a ligand to enhance BBB-targeted drug delivery [11]. In preclinical models, GSH-PEGylated liposomes significantly improved methotrexate delivery to the rat brain, achieving a four-fold increase compared to non-targeted liposomes [220]. Similarly, GSH-PEGylated liposomes loaded with DOX (2B3-101) enhanced brain accumulation of the drug by five-fold, resulting in reduced tumor growth and prolonged survival in glioma-bearing mice [221,222]. Encouraged by these results, two clinical trials (NCT01818713 and NCT01386580) were initiated to evaluate the safety and efficacy of 2B3-101 in patients with brain tumors and brain metastases [223,224].

Zhuo et al. developed a GSH-responsive nanoprodrug (cRGD/PSDOX-Cur@NPs), in which a disulfide bond in the DOX prodrug enables selective drug release in the TME, thereby reducing systemic cardiotoxicity [225]. Additionally, curcumin inhibits P-gp/ABCB1, mitigating drug resistance. This system demonstrated synergistic antitumor effects in vitro and superior BBB penetration with targeted brain delivery in vivo, exemplifying how a single drug delivery platform can address three major challenges in GBM therapy: BBB/BTB penetration, the acidic TME, and drug efflux.

### 3.24. Adsorptive-Mediated Transcytosis

The observation that polycationic proteins such as protamine bind to bECs’ surfaces and penetrate the BBB led to the concept of utilizing positively charged molecules for drug delivery [226]. These molecules interact electrostatically with the negatively charged surface of bECs, triggering endocytosis via clathrin- or caveolae-mediated pathways [11]. This mechanism facilitates transcellular transport across the BBB, enabling drug delivery to the brain. The BBB is particularly suited for AMT due to its specialized transcytotic pathways, unique morphology, enzymatic properties, and high mitochondrial content in bECs [11]. AMT-based strategies involve the conjugation of the therapeutic agent to (1) cationized proteins (e.g., cationic albumins), (2) endogenous cationic proteins (e.g., histones, protamine), (3) lectins such as wheat germ agglutinin (WGA) that bind endothelial glycoproteins, (4) positively charged cell-penetrating peptides (CPPs), e.g., the transactivator of transcription (TAT). Most research has focused on cationic albumins and CPPs.

Cationized albumin–drug conjugates emerged from early studies demonstrating that chemically modified albumin and immunoglobulin G (IgG) undergo significant AMT and accumulate effectively in the brain [11,227]. Preclinical models have demonstrated enhanced brain accumulation of therapeutic agents, including but not limited to aclarubicin [228], PTX [229], methotrexate [230], a combination of ibrutinib and hydroxychloroquine [231], and gene therapy vectors [232]. Among these, Lu et al. encapsulated cytotoxic plasmid pORF-hTRAIL in cationic albumin-conjugated pegylated NPs (CBSA-NP) for glioma gene therapy in a mouse model [232]. Within 30 min of intravenous administration, CBSA-NP-hTRAIL was detected in both the normal brain and tumor, and repeated injections induced apoptosis, substantially delaying tumor growth. Similarly, aclarubicin-loaded CBSA-NPs in a rat glioma model achieved 2.7- and 6.6-fold higher tumor drug concentrations compared to non-targeted NPs and aclarubicin solution, respectively [228]. Despite promising results, no further CBSA-NP studies followed.

CPPs, a diverse class of amphipathic, cationic peptides and peptide derivatives, can penetrate cellular membranes, including the BBB. The concept originated from Banks et al., who demonstrated that the HIV-1 regulatory protein, Tat, efficiently crosses the BBB (0.49 μL/g/min) [233,234]. Various peptide–drug conjugates and fusion constructs have since been explored in preclinical brain tumor models. TAT-modified gold NPs outperform free DOX in BBB penetration, GBM targeting, and circulation time [235]. Recently, TAT was fused to the Src-inhibiting region of Cx43 (TAT-Cx43266–283), a tumor suppressor protein, exhibiting greater tumor cell selectivity and lower toxicity than dasatinib [236,237]. Human and mouse GSC studies demonstrated that TAT-Cx43266–283 had superior antitumor effects compared to TMZ and erlotinib [237]. Future studies should evaluate this CPP with additional therapeutic agents.

The CPP p28, derived from *Pseudomonas aeruginosa*, has been shown to selectively penetrate the intact BBB. Research demonstrated that p28 exhibits a BBB permeability coefficient of 5 × 10^−6^ cm/s [238], which is markedly higher than that of TMZ (2.67 × 10^−6^ cm/s) [239]. Notably, p28 surpasses the established permeability threshold of 3 × 10^−6^ cm/s, considered indicative of favorable BBB penetration for CNS drug candidates [240]. The CPP p28 enters bECs primarily through caveolin-mediated endocytosis. However, the precise mechanism by which p28 penetrates the BBB remains unclear, necessitating further investigation to fully elucidate its transcytotic pathway. Once it crosses the BBB, p28 preferentially localizes to tumor lesions, where it functions as a therapeutic agent by stabilizing both wild-type and mutant p53 [238]. Mechanistically, p28 binds to the p53 DNA-binding domain and prevents COP1-mediated ubiquitination, thereby inhibiting proteasomal degradation of p53 and enhancing its tumor-suppressive activity. This leads to increased p53 stability and activity, promoting the expression of downstream tumor-suppressive genes such as p21 and p27, ultimately resulting in G2/M cell cycle arrest and apoptosis [238]. A Phase I trial within the Pediatric Brain Tumor Consortium (PBTC) enrolled 18 children with recurrent or progressive CNS tumors who received intravenous p28. Seven patients achieved stable disease (7–61 weeks), three had partial responses (44–125 weeks), and one achieved complete response (139 weeks), with good treatment tolerance. Three patients remained alive at 158, 140, and 110 weeks post-therapy [241]. Importantly, no immunogenicity or cellular toxicity were observed. Further investigation of p28 is needed, particularly in patients with secondary brain tumors, as preclinical studies have demonstrated that p28 enhances the efficacy of DNA-damaging agents, such as radiation and TMZ in brain metastases [238].

AMT ligands improve drug accumulation in brain tumors, though their generally low binding affinity necessitates cumulative interactions for effective transport. However, AMT lacks specificity, as negatively charged membranes are ubiquitous across cell types [11]. This poses a major limitation for delivering toxic agents, increasing the risk of off-target effects. Additionally, AMT ligands are prone to lysosomal sequestration or endothelial cytoplasmic trapping, often leading to unacceptable toxicity profiles [11]. To improve targeting precision and minimize off-target effects, recent strategies in CPP design have adopted dual-targeting approaches that combine AMT with RMT by incorporating ligands specific to the BBB or BTB [242,243,244,245]. This combinatorial strategy enables efficient translocation across the BBB while concurrently enhancing accumulation within tumor tissue, offering a promising avenue for the selective delivery of therapeutics to brain tumors [242,243,244,245].

CPPsite 2.0 is a publicly available, manually curated database that compiles 1855 experimentally validated CPPs developed for a wide range of therapeutic and delivery applications [246]. As a manually curated resource, each entry is extracted from the experimental literature and reviewed by experts, ensuring high-quality, reliable annotations. The database includes detailed information for each CPP, such as amino acid sequence, structural modifications, physicochemical characteristics (e.g., charge, hydrophobicity), cellular uptake efficiency, and cargo type. While not all peptides in the database are specifically designed for brain delivery, CPPsite 2.0 provides a valuable resource for identifying and optimizing candidates with potential for BBB permeability or compatibility with NP-based delivery systems. In the context of AMT, such curated datasets can accelerate the selection of promising CPPs by enabling in silico screening based on structural or functional properties relevant to BBB penetration.

### 3.25. Receptor-Mediated Transcytosis

Since the discovery of RMT systems at the BBB, considerable research has focused on identifying receptors predominantly expressed on bECs, particularly within the microvascular capillary network [11]. The fundamental principle of RMT for drug delivery is to exploit receptors selectively expressed on bECs by using ligands that specifically bind these targets. To achieve therapeutic efficacy, the ligand must be conjugated to a drug molecule or a drug delivery system, allowing the receptor–ligand complex to undergo endocytosis and vesicular transport across the BBB. This strategy enables the controlled and efficient delivery of therapeutic cargo into the CNS, while minimizing systemic exposure and off-target effects. Candidate BBB receptors for RMT-based drug delivery should not only facilitate directional transport from the luminal to the abluminal side across bECs but also exhibit minimal expression in peripheral tissues to limit adverse effects. Although no receptor has yet proven ideal, several have emerged as promising candidates for RMT-based delivery, including transferrin receptor 1 (TfR1), insulin receptor (IR), and low-density lipoprotein receptor-related proteins (LRPs), all of which are abundantly expressed on the luminal surface of the BBB [11,247,248,249,250].

IR, which in certain models shows significantly higher BBB transcytosis efficiency than TfR1, was among the earliest targets explored for receptor-mediated delivery. Pardridge and colleagues demonstrated that a human insulin receptor monoclonal antibody (HIRMAb) binds with high affinity to brain capillaries and undergoes rapid transcytosis across the BBB in primates, supporting its potential as a ligand in “Trojan Horse” drug delivery systems [11,249]. In contrast, receptors such as integrins or interleukin-13 receptors, although relevant for tumor targeting, are poorly expressed in the cerebral vasculature and are therefore unsuitable for RMT-mediated brain delivery [11,249,250].

Compared to CMT or AMT, RMT offers several advantages, including improved molecular selectivity, enhanced transport efficiency, and the ability to accommodate larger therapeutic cargo [11]. These features have made RMT a particularly attractive strategy for drug delivery in neuro-oncology, driving sustained efforts to optimize receptor targeting and intracellular trafficking [11]. The following section of this review will highlight the most extensively studied receptors currently under investigation for enhancing therapeutic transport across the BBB in brain cancer.

### 3.26. Transferrin Receptor 1

TfR1 is by far the most extensively studied and experimentally validated target for receptor-mediated drug delivery across the BBB, and is regarded as one of the most promising candidates for clinical translation [251]. As a bidirectional transcytotic system, TfR1 facilitates the influx of holo-transferrin (Tf) (iron-bound Tf) from the blood into the brain and the efflux of apo-Tf in the reverse direction [252]. It is abundantly expressed on bECs and significantly overexpressed in gliomas—reportedly up to 100-fold higher than in normal human astrocytes—further supporting its value as a therapeutic entry point for neuro-oncological drug delivery strategies [253].

Multiple ligands targeting TfR1 have been proposed to exploit RMT across the BBB in the setting of brain tumors. These ligands include (i) Tf; (ii) anti-TfR1 antibodies; (iii) bispecific antibodies, with one arm binding TfR1 and the other recognizing a therapeutic payload; (iv) TfR1-targeting peptides, such as T7 (HAIYPRH) and T12 (THRPPMWSPVWP); (v) aptamers composed of single-stranded RNA or DNA oligonucleotides that selectively bind TfR1. Collectively, these approaches underscore the versatility of TfR1 as a gateway for CNS drug delivery, offering a promising framework for overcoming BBB constraints and enhancing therapeutic precision in neuro-oncology.

### 3.27. Transferrin Conjugates

Tf is an iron-binding glycoprotein with a molecular weight of approximately 80 kDa [254]. Due to its abundance, availability, and relatively low cost, Tf has become one of the most commonly used ligands for targeting NPs, constituting approximately 43% of such approaches in the literature [255]. Numerous preclinical studies have demonstrated the ability of Tf-conjugated NPs to successfully cross the BBB and deliver a wide range of therapeutic agents. Representative examples include daunorubicin [256], siRNA [257], PTX [258], cisplatin [259], docetaxel [260], resveratrol [261], zoledronic acid [262], DOX [263,264,265], and ASOs [266]. Notably, PEGylated Polyamidoamine (PAMAM) dendrimer-based Tf-conjugated NPs loaded with TMZ have also been developed [267]. These NPs were shown to successfully traverse the BBB, delivering TMZ specifically to the avascular regions of the tumor. This targeted delivery resulted in effective TMZ dosing within tumor cells, leading to potent cytotoxicity against glioma cells, particularly GSCs in a mouse brain with xenografts [267]. Interestingly, Tf-functionalized NPs were used to transport siRNA against human P-gp/ABCB1 (P-gp/ABCB1-siRNA) specifically to the BBB, resulting in temporary reduction in drug efflux and enhanced drug permeability in the CNS. Beyond their ability to improve siRNA permeability through the BBB two-fold, 96 h post-transfection, functionalized polymeric NPs successfully reduced P-gp/ABCB1 mRNA expression up to 52%, compared with nonfunctionalized systems. Subsequently, the permeability of the P-gp/ABCB1 substrate, rhodamine 123, through the human BBB model increased up to 27% [257].

Recently, Lam et al. evaluated the use of TfR1-targeted liposome NPs loaded with TMZ and the bromodomain inhibitor JQ1, an emerging and promising therapeutic target [268]. The designed drug delivery system accumulated in the endothelial walls of brain microvessels, traversed the BBB, and subsequently aggregated in the surrounding brain tissue. This resulted in increased DNA damage and apoptosis, which correlated with a 1.5- to 2-fold reduction in tumor burden across two distinct intracranial orthotopic glioma models. Notably, the therapeutic efficacy of the TfR nanocarrier was more pronounced in the U87MG model compared to the GL261 model, likely due to the approximately 1.4-fold higher expression of Tf receptors in U87MG cells relative to GL261 cells. These findings suggest the potential for screening surface receptor protein levels in glioma patients to enable a more personalized approach to ligand-targeted nanotherapy [268].

### 3.28. Transferrin Receptor 1-Antibodies

Given that Tf conjugates often compete with endogenous Tf for TfR1 binding under physiological conditions, alternative targeting strategies have been explored. Antibodies such as RVS10 and OX26, which bind to epitopes on the extracellular domain of TfR1 distal to the Tf-binding site, have been engineered to circumvent this limitation [269,270,271].

A preclinical study demonstrated that cisplatin-loaded PEG liposomes conjugated with the OX26 monoclonal antibody (TPL-CisPt) achieved 1.43 times higher cellular uptake than standard cisplatin-loaded PEGylated liposomes (PL-CisPt) and prolonged survival in a rat glioma model 1.7-fold [272]. While these findings indicate that OX26-functionalized NPs can enhance drug delivery, the magnitude of improvement remains modest, raising questions about the clinical relevance of this approach. Interestingly, conjugating OX26 to the surface of TMZ-loaded PLGA NPs reduced drug release compared to non-modified PLGA NPs [273]. This effect may be attributed to the OX26 monoclonal antibody obstructing water permeation and hindering drug diffusion.

The primary limitation of OX26 and RVS10 as NP conjugates is their high affinity for TfR1, which often results in the sorting of the TfR/ligand complex to lysosomal degradation, reducing drug delivery to the brain parenchyma [274,275]. However, this effect varies depending on antibody engineering strategies. Additionally, their bivalent binding to TfR has been suggested to result in receptor crosslinking, leading to the formation of larger TfR networks on the cell surface [276,277]. Crosslinking can, under certain conditions, slow endocytosis and promote intracellular retention, thereby reducing transcytosis across the BBB [278]. These transcytosis-limiting effects are concentration-dependent [278,279]. At low antibody concentrations, bivalent antibodies with high affinity for TfR1 are more effective at crossing the BBB, as receptor crosslinking is minimal [278]. However, at higher concentrations, monovalent or low-affinity antibodies are generally more effective for brain uptake, as they avoid the endocytic limitations associated with receptor crosslinking [279]. Supporting this, a study examining the use of TfR-targeting antibodies conjugated to gold NPs demonstrated that switching from a bivalent to a monovalent binding mode resulted in a several-fold increase in brain uptake [280].

A direct comparison between Tf conjugates and TfR-targeting antibody conjugates is needed to more accurately assess their relative effectiveness and clinical potential. Moreover, optimizing antibody affinity and valency remains critical, as high-affinity bivalent antibodies, such as OX26 and RVS10, may still face challenges related to lysosomal degradation and receptor crosslinking, ultimately reducing brain delivery efficiency. Developing monovalent or low-affinity antibodies thus represents a promising strategy to overcome these barriers and enhance transcytosis across the BBB.

Bispecific antibodies have also garnered interest. Zhao et al. investigated bispecific antibodies as standalone therapeutic agents by fusing a VEGF inhibitor with a low-affinity, monovalent TfR-targeting antibody (VEGF-Trap/moAb4) [281]. This construct avoids competition with Tf for TfR binding and has demonstrated considerable success, resulting in more than a 10-fold increase in VEGF-inhibitor delivery to the brain parenchyma. This delivery effectively inhibited angiogenesis in U-87 MG xenograft models. However, while anti-VEGF treatment inhibited tumor angiogenesis, it did not translate into substantial tumor growth inhibition. Consistent with previous studies, the vascular remodeling induced by anti-VEGF therapy led to modest reductions in tumor burden but simultaneously created a more hypoxic TME, which is known to promote tumor cell invasion into adjacent brain tissue [282]. Therefore, future research should prioritize evaluating combinations of VEGF inhibitors with other agents that provide complementary antitumor effects within the framework of receptor-mediated delivery.

In addition to bispecific antibodies as standalone agents, their application as NP conjugates has also been explored. A recent study developed a pH-responsive anti-TfR bispecific antibody platform (pH-PEG engagerTfR) that binds PEGylated liposomal DOX at physiological pH and releases it in acidic endosomes (pH 6.0) following TfR-mediated transcytosis. The pH-PEG engagerTfR platform demonstrated a 12.8-fold increase in transport efficiency compared to a negative control lacking functional targeting and a 9.4-fold improvement over a non-pH-sensitive version of the platform [283]. As a result, the pH-PEG engagerTfR-decorated PEGylated liposomal DOX achieved enhanced antitumor effects and extended survival in a human GBM orthotopic xenograft mouse model. This nanoplatform exemplifies how a single pharmacological innovation can simultaneously improve BBB penetration and enable effective drug release within the TME.

Although significant progress has been made in utilizing anti-TfR1 antibodies for improving intracellular trafficking and transcytosis across the BBB, these approaches face several challenges. These include potential immunogenicity, size-related limitations in penetration, and complex manufacturing processes that substantially increase production costs [284,285,286]. Moreover, despite these advancements, the absolute number of NPs successfully delivered to the brain parenchyma often remains limited relative to the injected dose (~0.1% to 1%) [287,288]. To mitigate these limitations, single-chain variable fragments (scFvs) targeting TfR1 have emerged as promising alternatives [278]. ScFvs are small antibody fragments consisting of the variable regions of both heavy and light chains linked by a short peptide linker. These fragments retain the antigen-binding specificity of full antibodies while offering advantages such as lower cost, faster large-scale production, easier modification, and potentially reduced immunogenicity [289]. Recent studies have highlighted the potential of scFvs in enhancing the treatment of brain tumors. For example, TMZ-loaded liposomes decorated with anti-TfR1 scFv significantly improved survival in mice with intracranial GBM tumors, while also reducing toxicity. A TMZ dose 2.7 times lower than the free drug proved four times more effective in controlling tumor growth in TMZ-resistant tumors, representing a ten-fold increase in TMZ efficacy when delivered via scFv-based nanomedicine [290]. Beyond its use for TMZ delivery, the same nanocarrier platform was adapted to deliver the *p53* tumor suppressor gene [291]. Restoration of *p53* function addresses a key vulnerability in GBM, where *p53* mutations occur in approximately 30% of primary and 65% of secondary tumors [292]. In intracranial U87-luc2 xenograft models, *p53* delivery significantly extended survival compared with TMZ alone. Mechanistically, *p53* re-expression enhances TMZ sensitivity, potentially by modulating MGMT expression, a known contributor to chemoresistance. Furthermore, repeated cycles of combination therapy further amplified therapeutic benefit. Encouraged by these preclinical results, SGT-53 advanced into clinical evaluation. However, a Phase II trial (NCT02340156) in patients with recurrent GBM was terminated prematurely due to insufficient participant accrual, limiting the ability to assess clinical efficacy [292]. This experience highlights both the considerable therapeutic potential of receptor-targeted gene delivery and the operational challenges of translating such strategies into practice.

### 3.29. Transferrin Receptor 1-Targeting Peptides

TfR1-binding peptides, which target alternative receptor sites without competing with endogenous Tf, have emerged as promising ligands for drug delivery [293]. Their low cost, stability, and ease of development have contributed to their growing prominence, accounting for approximately 38% of investigated TfR-targeting strategies [255]. Among the various peptides explored, T7 (HAIYPRH) and T12 (THRPPMWSPVWP) have been the primary focus of research [255,294,295].

Preclinical studies have demonstrated that functionalizing NPs with TfR-targeting peptides significantly enhances transcytosis across BBB models, enabling deep tumor penetration [294] and facilitating the delivery of diverse therapeutics for GBM. Examples include PTX [296,297], vinblastine [298], siRNA [299,300], seliciclib [301], a combination of DOX and hTRAIL-encoding plasmid (pORF-hTRAIL, Trail) [302], vincristine [303], and antisense microRNA oligonucleotides [304].

Most studies have relied on L-form peptides, which are highly susceptible to enzymatic degradation [255]. D-form peptides, particularly those with retro-inverso sequences, offer greater stability and enhanced receptor affinity [305,306,307]. Yu et al. recently developed pegylated bilirubin NPs conjugated with the D-T7 peptide for dual-drug delivery—one formulation encapsulating cediranib and the other PTX [307]. In glioma-bearing C6 mice, combination therapy with D-T7-conjugated NPs extended median survival to 53 days, a 23% improvement over NPs lacking the D-T7 peptide.

Aptamers have emerged as highly versatile ligands for targeted drug delivery. These single-stranded RNA or DNA oligonucleotides adopt defined three-dimensional structures, enabling them to bind with exceptional specificity and affinity to their molecular targets [308,309]. Unlike antibodies, aptamers can be chemically synthesized with high reproducibility, scalability, and cost-effectiveness. Their small size—5 to 10 times smaller than IgG antibodies—enhances tissue penetration, a critical advantage in overcoming the BBB [310,311]. For instance, a 34-nucleotide aptamer has demonstrated BBB penetration rates up to 100 times higher than antibodies, although its overall transport remains modest at ~12% of the injected dose [309,312].

A unique feature of aptamers is their reversible binding, which can be modulated by introducing complementary nucleic acid sequences that disrupt their structure. Aptamers are generally considered nonimmunogenic and well tolerated, even at doses 1000-fold higher than conventional antibody therapeutics [309]. However, conjugation with PEG, commonly used to enhance stability, has been associated with immunogenic responses that may interfere with aptamer function [309].

In GBM therapy, aptamer-functionalized NPs have shown significant potential. Researchers recently developed a nucleic acid NP encapsulating TMZ, functionalized with two aptamers: GS24, which binds to TfR, and AS1411, which facilitates nuclear targeting in glioma cells [313]. This dual-targeting approach ensures precise drug delivery to the nucleus, where TMZ exerts its cytotoxic effect. By positioning TMZ in proximity to MGMT, the nanocarrier effectively disrupts DNA repair mechanisms, amplifying the drug’s anticancer activity and overcoming resistance.

In a more recent development, Su et al. engineered a multifunctional nanodevice with a core–shell structure tailored for GBM treatment [314]. The outer shell, composed of GBM cell membranes functionalized with TfR-targeting aptamers, facilitates BBB penetration and tumor-specific binding. The core consists of hollow manganese dioxide (MnO_2_), designed to release two therapeutic agents in response to the TME: KKGKGQQ-tetraphenylethene (Pep-TPE) and small interfering RNA (siRNA). Upon reaching GBM cells, the enzyme transglutaminase 2 (TG2) triggers Pep-TPE aggregation, generating fluorescence for high-resolution tumor imaging with an enhanced signal-to-noise ratio. Simultaneously, the siRNA silences TG2 expression, promoting apoptosis and increasing chemosensitivity. Both in vitro and in vivo studies have demonstrated that this nanocomplex effectively crosses the BBB, delivers its therapeutic payload, and exhibits robust antitumor activity.

The use of TfR-targeting peptides and aptamers offers a promising avenue for more effective brain tumor treatment. However, challenges such as peptide stability, aptamer immunogenicity, and the scalability of these technologies must be addressed to facilitate clinical translation. Continued research and well-designed clinical trials will be crucial in determining their long-term therapeutic potential.

### 3.30. Ferritin-Based Nanoparticles for Transferrin Receptor 1-Targeted Drug Delivery

In addition to ligand-based strategies exploiting TfR1, ferritin has emerged as a unique nanocarrier platform for drug delivery across the BBB. Unlike Tf, which is used to functionalize drug delivery systems through receptor targeting, ferritin leverages its natural TfR1 affinity and its hollow protein architecture to directly encapsulate therapeutic agents [315].

**Figure 6:**
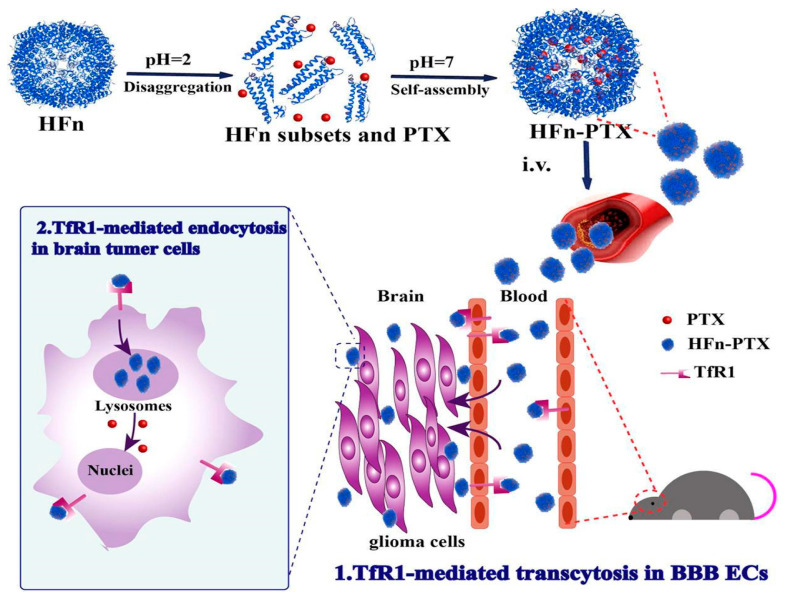
A schematic illustration of preparing an endogenous human ferritin heavy-chain nanocage (HFn) as a paclitaxel (PTX) carrier and its in vivo trafficking route. HFn-PTX can traverse the blood–brain barrier and enter glioma cells through TfR1-mediated endocytosis. Then, PTX is released in the lysosome and kills the host cells. Reused from Liu et al. [318].

Ferritin is a natural, spherical iron storage protein that, like Tf, binds to TfR1, although it interacts with distinct epitopes on the receptor. A study found that ferritin can traverse the BBB via TfR1-mediated transcytosis under physiological conditions, highlighting its potential for improving drug delivery across the intact BBB [315]. Composed of 24 subunits that self-assemble into a spherical shape, ferritin has a hollow core that can encapsulate a wide range of molecules, including therapeutic drugs and imaging agents [316,317]. This versatility has led to its exploration as a nanocarrier for the delivery of various therapeutic agents, such as DOX [317], PTX [318] (Figure 6), siRNA [319], topoisomerase I inhibitors [320], and immunotherapies [321], demonstrating significant therapeutic effects against preclinical glioma models, showing improved drug accumulation at the tumor site, reduced tumor growth, and prolonged survival. Wang et al. successfully delivered a non-nucleotide stimulator of interferon genes (STING) agonist, SR717, to orthotopic glioma models, eliciting a potent innate immune response via STING pathway activation. This activation initiated a signaling cascade that increased the production of type I interferons and proinflammatory cytokines, promoting T cell priming and enhancing infiltration of natural killer and dendritic cells. These effects led to a robust antitumor immune response, thereby significantly inhibiting tumor growth and prolonging survival [321]. A recent study explored ferritin as a delivery vehicle for oxaliplatin (OXA) in orthotopic glioma mice [322]. Notably, drug penetration was first demonstrated using an in vitro BBB model established according to Zhou et al. [323] with a TEER value exceeding 200 Ω·cm^2^, indicative of restrictive barrier properties suitable for mimicking key aspects of the physiological BBB. After crossing this validated model and being internalized by TMZ-resistant glioma cells via TfR1-mediated endocytosis, OXA induced apoptosis and elevated intracellular hydrogen peroxide (H_2_O_2_) levels. Simultaneously, ferritin downregulated ferroportin 1 expression, increasing intracellular iron retention and amplifying the Fenton reaction, which triggered ferroptosis—an iron-dependent form of cell death characterized by lipid peroxide accumulation. Unlike apoptosis, to which GBM cells frequently develop resistance, ferroptosis exploits GBM’s heightened iron metabolism and vulnerability to oxidative stress, making it a promising therapeutic strategy. Beyond its cytotoxic effects, the OXA@ferritin (OXA@Fn) complex also modulated the glioma immune microenvironment. It promoted dendritic cell (DC) maturation, shifted glioma-associated macrophages (GAMs) toward the proinflammatory M1 phenotype, and activated cytotoxic T lymphocytes (CTLs). These changes reversed glioma-associated immunosuppression by reducing regulatory T cell (Treg) recruitment and suppressing immunosuppressive cytokines, including interleukin-10 (IL-10) and transforming growth factor-β (TGF-β), which are upregulated in TMZ-resistant gliomas. Consequently, OXA@Fn significantly inhibited tumor growth and invasion in orthotopic TMZ-resistant glioma models. These findings underscore the potential of ferritin-based nanocarriers not only as drug delivery systems but also as modulators of the TME, paving the way for novel combinatorial strategies in GBM therapy.

**Figure 7:**
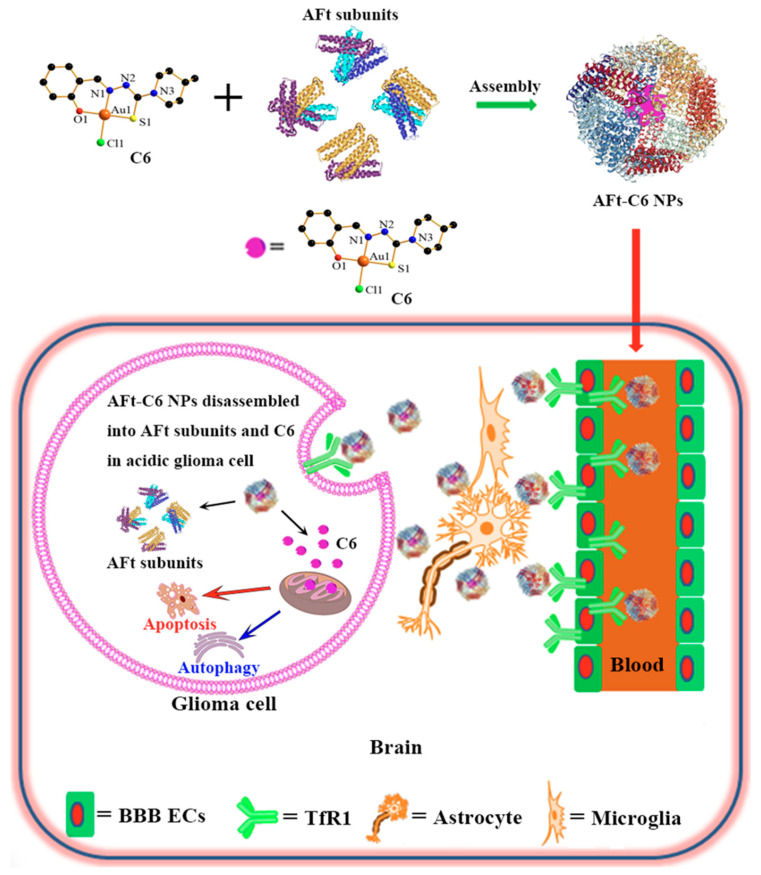
A scheme illustrating the development of a gold-based (Au) agent for glioma treatment using apoferritin nanoparticles (AFt NPs). The Au(III) 3-(4-methylpiperidine)thiosemicarbazide complex (C6) was encapsulated within AFt NPs and successfully delivered across the blood–brain barrier (BBB) in an orthotopic luciferase-expressing human glioma (Luc-U87MG) mouse model. Once in the acidic tumor microenvironment, C6 was released, inducing both lethal autophagy and apoptosis. Notably, only 5% of C6 was released at physiological pH (7.4), whereas 83% was released at acidic pH (4.7), demonstrating the stability of AFt-C6 in circulation and its selective disassembly within tumor tissue. Reused from Zhang et al. [324].

The iron-free form of ferritin, apoferritin, has also demonstrated promise as a delivery vehicle in preclinical glioma models. Notably, apoferritin has been used to deliver compounds such as gold(III) 3-(4-methylpiperidine)-thiosemicarbazides [324] (Figure 7), DOX [325], and vincristine sulfate [326], effectively penetrating mouse bECs (bEnd.3 cells) and accumulating within glioma tumor cells.

Both ferritin- and apoferritin-based nanocarriers offer several advantages, including excellent biocompatibility and biodegradability, owing to their composition of amino acids naturally occurring in humans [327,328,329]. They also enable high drug-loading efficiency and controlled release through TfR1-mediated endocytosis [315]. Additionally, they can be produced in large quantities through bacterial expression systems, such as *Escherichia coli*, making production cost-effective [327,328]. Furthermore, both systems are sensitive to acidic environments, enabling the release of encapsulated drugs upon entry into the acidic TME. Upon dissociation of the protein subunits, the hollow core is exposed, facilitating the controlled release of stored compounds [327,328]. Notably, apoferritin, owing to the absence of an iron core, provides a completely hollow internal cavity, offering greater capacity for the encapsulation of therapeutic agents compared to iron-loaded ferritin [327,328]. Moreover, apoferritin can undergo reversible disassembly into its 24 individual subunits under mildly acidic conditions (pH 2–3) and reassemble at neutral pH, enabling a straightforward and gentle method for cargo encapsulation without the need for extensive chemical modifications [327,328]. In contrast, native ferritin, due to the presence of its mineralized iron core, exhibits altered disassembly properties that complicate efficient drug loading.

### 3.31. Lactoferrin Receptor

Lactoferrin (Lf) is an iron-binding glycoprotein of the Tf family, known for its roles in immune defense, inflammation, and iron homeostasis [330]. Notably, Lf has been reported to cross the BBB via the lactoferrin receptor (LfR), which is expressed on bECs and is overexpressed in glioma cells, making it an attractive ligand for brain-targeted drug delivery [330]. Lf is also thought to interact with low-density lipoprotein receptor-related protein 1 (LRP1), another BBB-expressed receptor involved in transcytosis [11]. However, despite the receptor-mediated uptake observed in in vitro BBB models [11,330], direct in vivo studies demonstrate that endogenous Lf transport across the intact BBB is extremely limited, with brain uptake measured at only ~0.016%ID/g in rats [331]. This level of uptake is consistent with proteins largely confined to the brain’s plasma volume rather than achieving substantial parenchymal penetration [11]. As a result, while Lf-based strategies may enhance delivery under pathological conditions or within regions of BBB disruption, their intrinsic capacity for efficient transcytosis across the healthy BBB appears limited.

Preclinical studies have nevertheless demonstrated that Lf-functionalized NPs exhibit superior brain accumulation compared to Tf-functionalized systems, with up to a two-fold increase in brain uptake in healthy mice [332]. This enhanced targeting efficiency is attributed to the low plasma concentration of endogenous Lf, which minimizes receptor saturation under physiological conditions [332]. Consequently, Lf-NPs have emerged as promising platforms for delivering a wide array of therapeutics [333], notably encompassing (i) chemotherapeutics such as DOX [334,335,336,337], docetaxel [338,339], TMZ [340], and etoposide [341]; (ii) combination therapies, including carmustine with tamoxifen [342], simvastatin plus fenretinide [343], and inhibitors of apoptosis proteins (AZD5582 and SM-164) [344]; (iii) natural compounds such as etrandrine [345], shikonin [346], and curcuminoids, either alone [347] or combined with DOX [348]; (iv) gene therapy approaches, such as aurora kinase B siRNA [349].

To further refine targeting efficiency, Lf has been incorporated into dual-functionalized NPs [349,350,351]. One approach combined Lf with an RGD tripeptide, which binds integrin αvβ3, a receptor overexpressed on glioma cells. This strategy facilitated the preferential accumulation of docetaxel-loaded liposomes in GBM models, significantly prolonging survival in orthotopic glioma-bearing mice while minimizing systemic toxicity [349]. A distinct dual-targeting system combined Lf with an anti-CD133 antibody to address GSCs, which are implicated in tumor recurrence and therapeutic resistance. Encapsulating TMZ within this Lf/CD133-targeted lipid carrier demonstrated high specificity for glioma cells, achieving robust antitumor effects in vitro while exhibiting minimal toxicity toward normal cells [350].

Beyond serving as a targeting ligand, Lf has been explored as a primary structural component of NPs. Kumari et al. pioneered the development of TMZ-loaded Lf NPs, which achieved a three-fold increase in brain drug concentration, a five-fold increase in area under the curve (AUC), and a two-fold increase in drug half-life compared to free TMZ [352]. These NPs exhibited high TMZ entrapment efficiency (~45%), an optimized particle size (<160 nm), reduced opsonization—thereby extending circulation time—and pH-responsive drug release, with a four-fold increase in TMZ release under acidic conditions, potentially due to pH-dependent structural alterations in Lf [353]. Given that heparin has demonstrated anti-angiogenic effects in GBM, Hwang et al. developed an Lf–heparin conjugate that effectively inhibited tumor angiogenesis in an orthotopic GBM model [354].

Lf-based vehicles have also been incorporated into ferroptosis-inducing therapies, which exploit iron-dependent oxidative cell death. Ferroptosis is suppressed in GBM by the GPX4–GSH axis, which protects tumor cells from lipid peroxidation. Wang et al. developed a DDC/Cu–Fe complex encapsulated within Lf-conjugated albumin hybrid NPs (Alb/Lf NPs) to inhibit the cystine/glutamate antiporter (system Xc−) and deplete GSH, thereby disrupting GPX4 activity, inducing ferroptosis, and activating antitumor immunity, ultimately prolonging survival in glioma-bearing mice [355]. Similarly, Liang et al. co-assembled dihydroartemisinin (DHA), an iron-reactive antimalarial, with the photosensitizer indocyanine green (ICG) into an NP platform surface-modified with Lf to enhance brain targeting and ferroptotic activity [356]. In these models, the suppression of orthotopic GBM progression and a significant prolongation of survival were reported. Overall, while Lf-based NPs show considerable promise for enhancing drug delivery to glioma, particularly in the context of BBB disruption or tumor-induced permeability changes, their capacity for efficient transcytosis across an intact BBB appears limited, warranting further investigation.

### 3.32. Low-Density Lipoprotein Receptor Family

Low-density lipoprotein receptor (LDLR) and LRP1 are significantly overexpressed in both primary and secondary brain tumors, where increased demand for LDL provides the energy necessary for uncontrolled tumor growth [357,358]. GBM exhibits notably higher LDLR expression compared to low-grade astrocytoma and high-grade astrocytoma epidermal growth factor receptor variant III (EGFRvIII), a common epidermal growth factor receptor (EGFR) mutation in the mesenchymal GBM subtype, has been shown to significantly upregulate LRP1 expression, suggesting a correlation between elevated LRP1 levels and the poor prognosis associated with these tumors [359].

Both LDLR and LRP1 have been explored as targets for drug delivery in brain tumors. While LDLR expression at the BBB has been identified in an in vitro model [357], immunohistochemical analyses have failed to detect LDLR at the microvascular endothelium [360], aligning with the absence of LDL-bound cholesterol transport from blood to brain [361]. Nevertheless, several strategies have been developed to target what is presumed to be LDLR on the luminal surface of the BBB, despite the lack of direct evidence confirming its functional role in brain uptake. Given the limitations of using endogenous ligands such as LDL or apolipoproteins (ApoB, ApoE), including challenges in large-scale isolation and their tendency to aggregate, synthetic apolipoprotein-derived peptides—such as ApoB-BD (a 38-amino acid peptide) and various ApoEBD peptides (9–23 residues)—have been developed to retain LDLR-binding affinity while enhancing stability and scalability [362,363]. Sorafenib-encapsulating micelles coated with ApoE peptides enhanced anti-GBM activity 10.6- and 12.9-fold, and GBM accumulation 6.0- and 2.5-fold, compared to free sorafenib and non-targeted micelles, respectively [364]. Similarly, conjugated gold NPs with the ApoB29 peptide enabled efficient BBB penetration and were selectively taken up by microvascular bECs and pericytes in the GBM microenvironment, boosting proton therapy efficacy by 67–75% over proton therapy alone [365]. However, the potential risk of competition with endogenous ligands and potential disruption of CNS cholesterol homeostasis remains a concern. Peptide-22, a 22-amino-acid synthetic peptide mimicking an apolipoprotein A-I cholesterol-transporting domain, bypasses these limitations by binding LDLR without competing with endogenous LDL [366]. It offers high stability, low immunogenicity, and cost-effective synthesis. PTX-loaded NPs coated with PNP demonstrated superior glioma targeting and reduced off-target distribution, with glioma-to-brain fluorescence ratios of 3.95 versus 2.17 for non-coated NPs [367]. The same peptide improved resveratrol-loaded PLA NP transport across the BBB [368]. Separately, two distinct LDLR-targeting peptides, AGBBB015F and Regulon, were developed, both enhancing PTX-loaded NP uptake in U-87 MG glioma cells and a bovine BBB model [369]. However, despite these advances, the lack of definitive evidence for LDLR expression at the BBB raises critical questions about the viability of this approach.

LRP1 is a key transporter on the BBB and an attractive target for drug delivery due to its rapid internalization (t½ < 30 s compared to LDLR’s longer cycle of 10–15 min) [370,371]. While various natural and synthetic ligands have been explored for LRP1-targeted delivery, Angiopep-2 (ANG-2), a peptide derived from aprotinin, has received the most attention. While ANG-2 is widely proposed to facilitate BBB penetration via LRP1, definitive evidence supporting this pathway remains unresolved [11]. Binding studies demonstrate that ANG-2 engages with the CR56 and CR17 domains of LRP1, with CR56 being pivotal for receptor recycling and transcytosis [11,372]. Despite these interactions, ANG-2 exhibits minimal to undetectable affinity for LRP1’s primary high-affinity binding sites (II or IV), and inhibition studies reveal only partial reductions in uptake [11,373,374]. This raises critical questions regarding the prevailing model of LRP1-targeted drug delivery and points to the potential involvement of alternative pathways. To establish the role of LRP1, further studies utilizing high-resolution structural analyses and endothelial-specific knockout models are necessary. Until such data emerges, ANG-2’s classification as an LRP1-targeting peptide remains a tentative hypothesis [11].

Given its ability to penetrate the intact BBB and selectively accumulate in glioma cells, Angiopep-2 was leveraged for drug delivery through the development of ANG1005, a peptide–drug conjugate involving three PTX molecules. Preclinical studies demonstrated a ~161-fold greater brain uptake of ANG1005 compared to free PTX within 30 min of intravenous injection [375]. These promising results led to clinical trials, positioning ANG1005 as the first RMT-based chemotherapeutic strategy to reach clinical evaluation. A Phase II trial in breast cancer patients with leptomeningeal disease reported a 79% intracranial disease control rate and a median overall survival of 8.0 months (95% CI, 5.4–9.4), significantly exceeding the historical median survival of 2–4 months [376]. These findings prompted a large, randomized Phase III trial (NCT03613181) in HER2-negative breast cancer patients with newly diagnosed leptomeningeal disease or previously treated brain metastases. In gliomas, ANG1005 demonstrated BBB penetration and therapeutic tumor concentrations with an acceptable safety profile. A Phase I study showed complete response in one patient and partial responses in two others at doses of 420–700 mg/m^2^, while 30% of patients at 420 mg/m^2^ achieved stable disease for a median of 51 days [377]. A subsequent Phase II study (NCT01967810) assessing 600 mg/m^2^ in recurrent HGGs reported a clinical benefit rate of 46.6%, increasing to 53.6% in anaplastic gliomas [378]. However, efficacy was limited in bevacizumab-refractory GBM, and overall survival gains were modest. Notably, patients with high SSR3 expression had a median overall survival (OS) of 18 months versus 9 months in those with low SSR3 expression, suggesting a potential biomarker-driven therapeutic approach. At present, there are no plans for clinical trials involving ANG1005 [378].

Angiopep-2 has also been extensively studied as a targeting ligand for NP-based drug delivery. Preclinical studies have demonstrated its ability to effectively deliver various agents, including irinotecan [379], TMZ both alone [380] and in combination with cisplatin [381] and gene therapy [382], docetaxel [383], PTX [384], arsenic trioxide [385], carmustine [386], DOX both alone [387] and in combination with siRNA [388], phytochemicals [389], a combination of small interfering RNA (siYAP) with verteporfin [390], PI3K/Akt inhibitors [391], and gene therapies [392,393]. Collectively, preclinical studies highlight Angiopep-2’s promise for brain tumor drug delivery. Nevertheless, unresolved questions regarding its exact mechanism of BBB transport, alongside challenges in manufacturing scalability and pharmacokinetic optimization, remain significant barriers to clinical translation. Further development of more potent and specific LRP1-targeting ligands will be essential to fully realize the therapeutic potential of RMT strategies.

### 3.33. Nicotine Acetylcholine Receptors

NAChRs are widely expressed in the CNS, and their significant upregulation in GBM cells has drawn attention to their potential as targeting ligands for drug delivery strategies [394]. Peptide neurotoxins and viral proteins that interact with nAChRs have been reported to cross the BBB, fueling interest in leveraging nAChRs for RMT [395]. Among the various subtypes, the α7 nAChR has been primarily explored due to its proposed involvement in modulating BBB properties [396]. However, the expression of functional nAChRs on bECs remains a matter of debate. In vivo immunohistochemical studies have largely failed to detect nAChRs on bECs [397], and much of the supporting evidence stems from in vitro models, which may not fully recapitulate the complexity and cellular interactions present in the native BBB [11,398]. This discrepancy underscores the need for cautious interpretation of preclinical findings and highlights a critical gap that must be addressed before clinical translation.

Despite these uncertainties, several ligands targeting nAChRs have been developed to enhance CNS drug delivery. For example, DCDX (FKESWREARGTRIERG), a 16-amino-acid peptide, has been shown to cross the BBB in vitro and in vivo, presumably via interaction with α7 nAChRs [399]. However, direct in vivo evidence confirming receptor-specific engagement remains limited. Han et al. demonstrated that DCDX-functionalized liposomes encapsulating DOX, when co-administered with the P-gp/ABCB1 inhibitor verapamil, significantly extended survival in a murine GBM model—from a median of 25 to 37 days [399]. Notably, the internalization of DCDX-decorated nanocarriers appears to involve multiple endocytic pathways—including lipid raft/caveolae-mediated, clathrin-dependent, and actin cytoskeleton-mediated mechanisms—suggesting that uptake may not be solely reliant on receptor binding [399]. Further mechanistic studies are required to delineate the relative contribution of α7 nAChR engagement versus nonspecific endocytosis to the observed BBB transcytosis.

Another extensively studied ligand, RVG29, is a 29-residue peptide derived from the rabies virus glycoprotein [400], previously used to transport siRNA [401] and small molecules like itraconazole across the BBB [402]. Recent advances have incorporated RVG29 into a multifunctional nanoplatform composed of iron oxide NPs (Fe_3_O_4_), chitosan, and Zeolite Imidazolate Framework-8 (ZIF-8) [403]. In the acidic TME, ZIF-8 disintegrates, releasing Zn^2+^ and generating ROS that trigger caspase-9-dependent mitochondrial apoptosis. Notably, the Fe_3_O_4_ core facilitates MRI-guided localization and, under exposure to an alternating magnetic field (AMF), significantly accelerates tumor cell destruction, with a 30 s AMF exposure leading to >89% GBM cell death—achieving in seconds what takes 48 h without AMF—while sparing healthy brain tissue [403].

RVG29 has also been utilized to deliver TMZ via NPs formed from zein, a biocompatible plant-derived protein with high drug-binding capacity and minimal cytotoxicity. TMZ@RVG-Zein NPs accumulated effectively in U87 GBM cells in vitro, releasing TMZ and exerting antitumor effects [404]. In an effort to reduce molecular weight and optimize NP size and cost, a truncated variant called RVG15 was developed. Despite having only 15 amino acids, RVG15 retains equivalent brain-targeting efficacy and has been successfully used to deliver DOX-loaded nanomicelles [405] and PTX-loaded liposomes [406], showing significant therapeutic benefits in both in vitro and in vivo models.

Inspired by the high affinity of snake venom-derived peptides for nAChRs, Zhan et al. engineered KC2S, a synthetic peptide resembling the loop 2 region of three-finger neurotoxins [407]. KC2S displays similar nAChR-binding affinity to RVG29 but with a smaller peptide backbone. When used to functionalize PTX-loaded PEG-PLA micelles, KC2S significantly prolonged survival in mice bearing intracranial GBM xenografts.

Nevertheless, while these findings are encouraging, critical questions remain regarding the precise role of nAChRs—particularly α7—in mediating BBB/BTB transcytosis in vivo. Clarifying receptor expression patterns on bECs and distinguishing receptor-mediated uptake from nonspecific mechanisms are essential next steps toward optimizing and validating nAChR-targeted delivery platforms for clinical applications.

### 3.34. Growth Factor Receptors

Insulin-like growth factor receptor (IGF-1R) is expressed on the BBB, and is reported to be approximately twice as abundant as TfR in isolated brain microvessels [408,409]. Beyond the BBB, IGF-1R is also expressed in glioma cells [410] and brain metastases [411], where it contributes to tumor proliferation, survival, and therapeutic resistance. IGF-1R binds IGF1 with high affinity and IGF2 and insulin with lower affinity, and is capable of delivering its ligands to specific brain regions, including the hypothalamus and hippocampus [409]. Upon ligand binding, IGF-1R undergoes internalization via caveolin- or clathrin-dependent pathways [409]. In vivo, the transport of circulating IGF1 across the BBB is regulated by local neuronal activity [409]. Recently, IGF1R5, a camelid single-domain antibody (VHH) targeting IGF-1R, has been shown to cross the BBB and deliver neuroactive peptides, such as galanin and neurotensin, which typically do not penetrate the BBB [412]. Similarly, quantitative assessments revealed that up to 25% of the administered IGF-1R sdAb-fusion proteins localized within the brain parenchyma within a brief 5 min perfusion period, underscoring the rapid and substantial transcytosis capability of this system [409]. Future studies should explore the potential of IGF-1R-targeted ligands as delivery vehicles for therapeutic agents against brain tumors, given their demonstrated ability to efficiently and rapidly transport biologics across the BBB.

EGFR, located on the luminal side of the BBB, plays a key role in the infiltration of cancer cells and the facilitation of brain metastasis [413]. EGFR, along with its mutant variant EGFRvIII, is overexpressed in GBM cells, neovasculature, VM, and GSCs [413,414]. EGFR is estimated to be overexpressed in approximately 60% of primary GBMs [414]. As a result, EGFR has been extensively studied as a target for enhancing drug delivery using various ligands. Kuo et al. demonstrated that anti-EGFR antibodies conjugated to cationic solid lipid NPs encapsulating carmustine achieved 80% cell death in U87MG cells [415]. Mamot et al. delivered chemotherapeutics, including DOX and vinorelbine, via modified cetuximab fragments targeting both wild-type EGFR and EGFRvIII, achieving up to 80% cell death in glioma cells within 15 min [416]. Last but not least, researchers conjugated TMZ-loaded PLGA NPs with panitumumab (PmAb), an anti-EGFR antibody, resulting in superior cytotoxic effects in EGFR-overexpressing U-87 MG cells [417].

EGFR-specific peptides and aptamers have also shown promise. Mao et al. developed a d-peptide ligand with high affinity for EGFR and EGFRvIII, conjugated to PTX-loaded micelles, which exhibited improved transcytosis and penetrating capabilities in a BTB/U87 tumor spheroid co-culture model [414]. The nanosystem demonstrated superior antitumor effects compared to PTX. Furthermore, recent studies have explored EGFR-specific DNA aptamers, such as GR20hh, to deliver DOX into patient-derived GBM cells [418].

Vascular Endothelial Growth Factor Receptor 2 (VEGFR-2) is overexpressed in GBM cells, playing a crucial role in tumor angiogenesis and metastasis [419]. VEGFR-2 binds to neuropilin-1 (NRP-1), which enhances VEGF-A binding, amplifying angiogenic signaling [420]. Both VEGFR-2 and NRP-1 promote endothelial cell proliferation, migration, and the formation of vasculogenic mimicry [87]. Although VEGFR-2 expression is absent on the luminal side of the BBB, targeting VEGFR-2 could be valuable in dual-targeting strategies where a receptor on the BBB is also targeted [11,420].

DA7R, a peptide that specifically binds to both VEGFR-2 and NRP-1, is resistant to enzymatic degradation. It has been shown to competitively inhibit VEGF binding to NRP-1, providing anti-proliferation and anti-angiogenesis effects. Researchers have successfully encapsulated carfilzomib, a proteasome inhibitor, in lipid-based nanodisks conjugated with DA7R [421]. This nanosystem demonstrated improved tumor accumulation, suppressed tumor growth, and extended survival in intracranial GBM models. Similarly, DOX-loaded liposomes conjugated with DA7R achieved an 82% inhibition of angiogenesis in a GBM cell model [421]. Dual-targeted liposomes modified with DA7R and T7 peptides (TfR1-targeting) enhanced tumor delivery of DOX and vincristine, exhibiting improved antitumor effects compared to free drugs [422]. Additionally, bevacizumab-modified nanostructured lipid carriers delivering docetaxel reduced tumor volume by 70% and enhanced docetaxel’s antitumor effect 1.6-fold compared to the free drug [423].

### 3.35. Folate Receptor

Researchers have explored the idea of using folic acid as a ligand due to its transport across BBB. There is evidence for expression at the BBB of both the folate receptor (FOLR1), which is an RMT system, and the reduced folate carrier (FRC), which is a CMT system [11]. II FOLR1 is indeed the primary folate transporter at the BBB, then folate-conjugated NPs are likely to traverse the barrier via RMT, facilitating their entry into the brain parenchyma [11]. Conversely, if FRC is the principal folate transporter, the ability of folate-conjugated NPs to penetrate the BBB may be limited by the restrictive nature of the CMT system, with its narrow transport channels potentially obstructing efficient transcytosis [11]. So far, researchers have developed docetaxel- and ketoconazole-loaded folate-grafted solid lipid NPs, reporting that the brain permeation coefficients (Kin) of the nanosystems were 44 times higher than that of free docetaxel [424]. Furthermore, a modified nanodrug delivery system based on polyhedral oligomeric silsesquioxane (POSS) with folic acid and iRGD peptide molecules was reported to improve the accumulation of TMZ in GBM-bearing mice, leading to a considerable increase in survival. The iRGD peptide was specifically used to enhance the penetration of NPs across the BBB and into solid tumors by binding to integrins (such as αvβ3 and αvβ5) overexpressed on bECs. The use of folic acid as a ligand is indeed a promising strategy for improving drug delivery across the BBB [425]. However, elucidating the precise transport mechanisms, including the roles of FOLR1 and FRC, is crucial in order to support further research and optimize the efficacy of folate-conjugated NP systems.

### 3.36. Leptin Receptor

The leptin receptor (LEPR) is prominently expressed on the luminal surface of the BBB, CSF barrier, and choroid plexus, where it plays a pivotal role in regulating food intake and metabolism [425]. Notably, LEPR overexpression has been implicated in the progression of GBM, where it promotes tumor growth and endothelial-cell-dependent angiogenesis. Given its substantial molecular weight of 16 kDa, leptin is incapable of passive diffusion across the BBB, thereby necessitating the engagement of RMT mechanisms [11,426]. Upon activation of LEPR, leptin traverses the BBB via transcytosis, underscoring the promise of exploiting LEPR for targeted drug delivery strategies aimed at the brain.

Various leptin-derived peptides have emerged as promising brain-targeting ligands. Among these, the 30-amino-acid peptide leptin30 shares similar brain accumulation efficiency with the native hormone, positioning it as a potentially effective tool for targeted drug delivery to the CNS [427]. Recent studies have demonstrated the successful conjugation of leptin30 to dendrigraft poly-L-lysine (DGL)-PEG nanocarriers loaded with DNA, resulting in effective transcytosis across in vitro BBB models and significant gene transfection efficiency both in vitro and in vivo [428]. More recently, a novel leptin-derived peptide, LDP 14, has been developed, demonstrating superior internalization efficiency in human bECs and U87 glioma cells when compared to leptin30 [429]. In vivo experiments confirmed the potential of LDP-14-modified NPs, showing efficient brain accumulation and reduced biodistribution in peripheral organs. Targeting LEPR with leptin-derived peptides offers a promising strategy for enhancing drug delivery across the BBB. Although early results are promising, further understanding of transport mechanisms and optimization of peptide-based nanocarriers are essential for clinical translation.

### 3.37. Scavenger Receptors

Scavenger receptors (SRs) are a diverse family of cell surface glycoproteins expressed across a range of cell types, including macrophages, dendritic cells, and endothelial cells [430]. These transmembrane and soluble proteins can bind a wide array of both endogenous and exogenous ligands. The SR family consists of eight distinct classes (A–H), each encoded by unrelated genes. Notably, SR-B (also known as CD36) is localized to the microvasculature in the brain, while SR-A is expressed by microglia and perivascular cells [430]. Increasing evidence has elucidated the role of SRs in mediating the uptake of complexes across the BBB. For example, SR-B is a receptor for oxidized low-density lipoprotein (LDL) and acetylated LDL, while SR-A specifically binds to spherical nucleic acids (SNAs) [430].

In a groundbreaking study, SNA-NPs conjugates, loaded with siRNA targeting *Bcl2Like12* (*Bcl2L12*), a known p53 inhibitor overexpressed in GBM, were shown to successfully cross the BBB after systemic administration [431]. These nanodrug systems selectively accumulated in tumor tissue and disseminated throughout the glioma, leading to enhanced apoptosis in glioma-bearing mice without causing significant side effects. Importantly, the inhibition of SR-A using poly I and fucoidan effectively blocked SNA uptake into glioma cells and transcytosis across the BBB, thus confirming the critical role of SR-A in mediating NPs delivery across the BBB.

This study laid the groundwork for a Phase 0 clinical trial (NCT03020017) which assessed the safety and efficacy of gold NP-based SNAs conjugated with siRNA specific to Bcl2L12 in patients with recurrent GBM or gliosarcoma [432]. The intravenously administered SNA demonstrated an ability to cross the BBB/BTB effectively, accumulating in GBM cells and reducing the target Bcl2L12 protein expression. These results underscore the potential of SNA-based delivery systems as a novel therapeutic strategy for GBM, offering promise for improved treatment efficacy through targeted delivery to brain tumors.

Although receptor-targeted drug delivery systems for brain tumors have advanced considerably, critical challenges remain. Tumor heterogeneity, both between and within lesions, necessitates a shift towards personalized strategies informed by receptor profiling to maximize targeting efficacy.

While RMT offers a promising non-invasive strategy for delivering therapeutics across the intact BBB, its clinical translation remains limited by several intrinsic challenges. A key limitation is that most RMT targets, including TfR1 and LRP1, are also expressed at significant levels in peripheral tissues such as the liver, spleen, and kidneys. This off-target expression can lead to systemic distribution of the therapeutic payload, diluting brain specificity and increasing the risk of peripheral toxicity. Emerging dual-targeting strategies that utilize one ligand to engage receptors on the BBB for efficient transcytosis, combined with a second ligand targeting tumor-specific receptors, hold great promise to improve delivery selectivity, enhance tumor penetration, and reduce off-target accumulation and systemic toxicity. Other challenges include receptor saturation, which occurs when high doses overwhelm available receptors, competition with endogenous ligands for receptor binding, and variability in receptor expression among individuals, which may result in inconsistent treatment outcomes, complicating the prediction and optimization of therapeutic efficacy across patient populations. Beyond BBB transcytosis, therapeutic success also depends on overcoming intracellular barriers such as endosomal escape, lysosomal degradation, and controlled release within the TME. Emerging strategies that combine multimodal or sequential targeting may improve tissue specificity and enhance the therapeutic index. However, successful clinical translation will require the careful consideration of safety, immunogenicity, manufacturability, and regulatory compliance. Continued interdisciplinary collaboration at the intersection of nanotechnology, neuro-oncology, and vascular biology will be essential to unlocking the full potential of RMT-based delivery systems for patients with brain tumors.

### 3.38. Cell-Mediated Drug Delivery

The concept of cell-mediated drug delivery across the BBB leverages cells as biological vehicles, offering distinct advantages over conventional drug delivery methods, including enhanced biocompatibility, prolonged circulation times, and reduced toxicity. Mesenchymal stem cells (MSCs), neural stem cells (NSCs), and macrophages have been the primary cellular carriers investigated for improving drug delivery in neuro-oncology [11].

### 3.39. Mesenchymal Stem Cells

While there are reports suggesting that MSCs derived from bone marrow (BM-MSCs), adipose tissue (AD-MSCs), and umbilical cord blood (UCB-MSCs) have the potential to cross the BBB and could thus serve as tools for CNS drug delivery, the evidence remains inconclusive [11]. It is important to note that no definitive or consistent proof exists that MSCs can naturally penetrate an intact BBB under normal physiological conditions [11]. Studies cited as evidence for MSC transport across the BBB often fail to substantiate this claim [11,433,434]. For instance, one study involved the direct injection of MSCs into the spinal cord, bypassing the BBB entirely. In another study, MSCs were intravenously administered following spinal cord injury, a condition in which the blood–spinal cord barrier is compromised for an extended period [433]. Early investigations found no MSCs in the brain parenchyma after intravenous injection; however, they were observed in the meninges, which lack a BBB. These observations suggest that MSCs do not cross the intact BBB [435]. Even in pathological conditions that disrupt the BBB, such as stroke, only a minimal fraction (approximately 1%) of injected MSCs successfully reach the ischemic brain [436].

### 3.40. Neural Stem Cells

NSCs, multipotent progenitor cells derived from both developing and adult CNS tissue, similarly to MSCs, do not efficiently cross the BBB under physiological conditions [437]. Even in the context of pathological BBB disruption, their transmigration remains limited [437,438]. However, NSCs exhibit pronounced tumor-tropic properties, which make them potential tools for improving tumor targeting, provided that the BBB/BTB is bypassed (e.g., through intratumoral injection). In rodent models of intracranial gliomas, NSCs implanted into the brain have been shown to infiltrate the tumor core, extend along the invasive margins, and migrate through the brain parenchyma—even when introduced at sites distant from the tumor [437]. Intravenous administration via the tail vein demonstrated limited homing to brain tumors, with accumulation restricted to areas of BBB breakdown, while sparing healthy brain regions with intact barrier function. These observations have supported the exploration of NSCs as delivery vehicles capable of targeting disseminated tumor foci and delivering therapeutic payloads to residual, infiltrative disease—an especially compelling strategy in the treatment of highly diffuse tumors such as GBM. For instance, in a clinical trial (NCT01172964) involving patients with recurrent HGGs, NSCs genetically engineered to express the prodrug-converting enzyme cytosine deaminase (CD) were administered intracranially at the time of tumor resection or biopsy [439]. Following implantation, patients received oral 5-fluorocytosine (5-FC), a prodrug converted by CD into the active chemotherapeutic agent 5-fluorouracil (5-FU) directly within the tumor. This strategy aimed to enhance local drug concentrations while minimizing systemic exposure and toxicity to normal brain tissue and peripheral organs. CD-expressing NSCs successfully migrated to distant tumor sites. Notably, 5-FU was detectable in the brain tissue of all patients, but not every patient had detectable levels in plasma. Given the limited ability of systemically administered 5-FU to penetrate the CNS, the consistently higher concentrations of 5-FU in brain tissue relative to plasma strongly suggest that the intratumoral drug levels resulted primarily from localized enzymatic conversion by CD-expressing NSCs. In a separate clinical trial (NCT03072134), NSC-CRAd-S-pk7, an engineered oncolytic adenovirus delivered by NSCs, was administered directly to the resection cavity walls in patients with newly diagnosed HGGs [440]. This strategy triggered an immune-mediated anti-glioma response, particularly in patients with unmethylated MGMT promoters. The trial reported a median progression-free survival of 9.05 months and overall survival of 18.4 months, with the treatment proving both safe and nontoxic. Currently, another clinical trial is exploring the delivery of oncolytic herpes simplex virus-1 (HSV) via NSCs in children with recurrent HGGs (NCT04482933).

### 3.41. Macrophages

Under physiological conditions, the BBB restricts the infiltration of peripheral immune cells into the CNS, primarily due to the low expression of leukocyte adhesion molecules (LAMs) on bECs [441,442,443]. In the context of inflammation—a key contributor to tumor initiation, progression, invasion, and metastasis—there is an upregulation of cellular adhesion molecules (CAMs), which facilitates the rolling, firm adhesion, and transendothelial migration of immune cells, including macrophages [441]. Simultaneously, hypoxic tumor regions secrete chemotactic factors that promote the recruitment and retention of macrophages within the TME [442,443]. As a result, macrophages emerge as the predominant immune cell population infiltrating GBM, comprising up to one-third of the tumor mass and playing a central role in sculpting its profoundly immunosuppressive landscape [444]. Similarly, these cells are among the most abundant immune populations within the brain metastatic niche [445].

**Figure 8:**
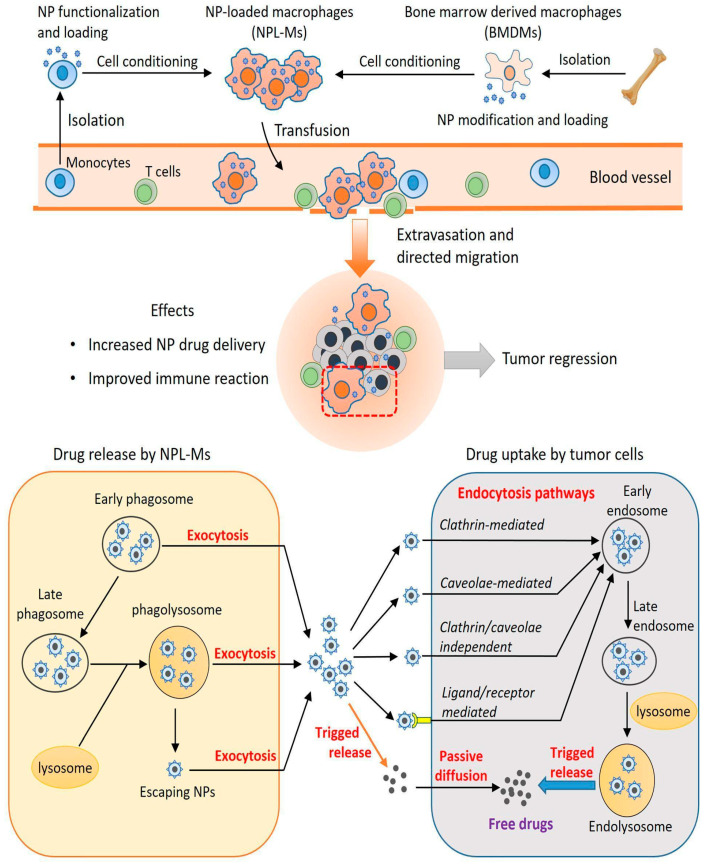
Macrophage-mediated nanoparticle (NP) delivery leverages live macrophages derived from peripheral monocytes, bone marrow, or established cell lines as cellular carriers. Following systemic administration, nanoparticle-loaded macrophages (NPL-Ms) migrate preferentially to tumor sites, where they are released via exocytosis, occurring either after recycling from early phagosomes and mature phagolysosomes or following escape from these intracellular compartments. Tumor cells internalize these NPs through multiple endocytic pathways, including clathrin-mediated, caveolae-mediated, and clathrin/caveolae-independent mechanisms. Surface modifications of NPs with specific ligands further facilitate receptor-mediated endocytosis, improving cellular uptake. Once internalized, NPs traffic through the endosomal–lysosomal system, where they can be triggered to release their drug payload. The liberated free drugs then diffuse passively across intracellular membranes, exerting their cytotoxic effects within tumor cells. Reused from Ding et al. [447].

Building on these insights, an emerging therapeutic strategy involves harnessing macrophages as cellular carriers capable of transmigrating across the BTB and homing selectively to tumor tissue. Notably, macrophages can preserve their drug-carrying capacity during this process, enabling the targeted delivery of therapeutics deep within the TME [446]. The therapeutic efficacy of macrophage-mediated NP delivery hinges on the controlled release of drug cargo from NP-loaded macrophages and efficient uptake by neighboring tumor cells. NPs may be released from macrophages via exocytosis, either following recycling from early phagosomes or phagolysosomes or through escape from intracellular compartments. Within the TME, NPs are internalized by cancer cells through multiple endocytic pathways, including clathrin-mediated, caveolae-mediated, and clathrin/caveolae-independent mechanisms. Upon internalization, NPs are trafficked through the endosomal–lysosomal system, where drug release is triggered. The liberated drug then diffuses passively across intracellular membranes to exert cytotoxic effects within tumor cells [447] (Figure 8). In preclinical models, murine macrophage-like cells incubated with DOX-PLGA-PEG-NPs (referred to as M-NPs) demonstrated superior tumor infiltration compared to free NPs [446]. Specifically, in 3D glioma spheroids, M-NPs penetrated 1.5-fold deeper than free NPs. In ex vivo brain sections, free NPs accumulated at the tumor periphery, whereas M-NPs exhibited widespread intratumoral distribution, supporting the notion that macrophages can overcome barriers such as the BTB and elevated IFP to enhance drug delivery.

Nonetheless, off-target accumulation remains a concern, as M-NPs were also detected in organs such as the spleen, kidneys, and lungs. To improve tumor selectivity and reduce off-target distribution, one proposed strategy involves coupling macrophages with ligands that engage RMT pathways.

Recent developments have explored alternative formulations to address these limitations. For instance, macrophages were loaded with ferritin-based protein nanocages encapsulating therapeutic agents. When administered intravenously or intratumorally, these constructs were well tolerated and conferred a survival benefit in two orthotopic murine glioma models (GL261 and CT-2A) [448]. To better understand how ferritin-based drug carriers move from macrophages to glioma cells, researchers conducted interactome studies, which revealed that phagocytic pathways and cytoskeletal rearrangement mechanisms are involved in the uptake of ferritin nanocages by macrophages and their subsequent transfer to tumor cells [448]. Another as-yet-unexplored strategy entails modulating the TME to enhance cellular carrier homing. The local administration of chemoattractants could establish chemokine gradients that preferentially attract carriers expressing cognate receptors [449].

Importantly, the rationale for employing macrophages as drug delivery vehicles is rooted not in their intrinsic ability to penetrate the BBB, but rather in their inherent tumor-homing properties. To harness this advantage while avoiding the practical limitations associated with using whole cells, Xu et al. engineered macrophage membrane-coated nanovesicles functionalized with the angiopep-2 peptide, thereby combining tumor targeting with enhanced transcytosis across the BBB [450]. These biomimetic nanocarriers achieved a 19.75-fold increase in accumulation of the anti-programmed death-ligand 1 (aPD-L1) antibody compared to the free antibody, resulting in complete tumor regression, prolonged survival, and the establishment of long-term immune memory in orthotopic GBM models.

### 3.42. Exosomes

The limited success of MSCs, NSCs, and macrophages in enhancing brain drug delivery has prompted increasing interest in exosomes as potential drug delivery vehicles [11]. Exosomes are small (30–100 nm) naturally occurring extracellular vesicles, secreted by cells via the plasma membrane, with the ability to be efficiently internalized by neighboring cells [451]. Typically isolated from cell cultures by removing cell debris through centrifugation, exosomes are of particular interest in drug delivery due to their biocompatibility and capacity to carry various bioactive molecules [451]

For exosomes to be effective in brain drug delivery, they require the incorporation of ligands that facilitate RMT across the BBB, as without such ligands, their capacity for BBB penetration is inherently limited [11]. While genetic modification of the producer cells offers a method to incorporate these ligands, an alternative strategy involves utilizing exosomes derived from cells that naturally express surface ligands capable of binding to specific receptors on the BBB, thereby enhancing their targeted delivery potential [11]. For example, exosomes derived from the SK-Mel-28 breast cancer cell line, which express CD46 (an inhibitory complement receptor), have been shown to penetrate an in vitro human BBB model (hCMEC/D3) [452]. Similarly, exosomes isolated from a murine macrophage cell line were shown to carry LFA-1 on their surface, which can interact with intercellular adhesion molecule-1 (ICAM-1), a receptor found on bECs. To further enhance targeting, macrophage-derived exosomes were modified with a tumor-targeting peptide, cRGD, which binds to integrin αVβ3 receptors on diffuse intrinsic pontine glioma (DIPG) cells [453]. This modification allowed the exosomes, loaded with panobinostat and PPM1D siRNA, to cross the BBB, accumulate at the tumor site, and be internalized by DIPG cells via cRGD, releasing their cargo through endosomal escape. This approach resulted in significant tumor growth inhibition and prolonged survival in orthotopic DIPG-bearing mice. Despite these promising advancements, exosomes face several obstacles to becoming an effective brain drug delivery system. These include their low yield from the starting cell line, rapid systemic clearance (similar to non-PEGylated liposomes), complex engineering requirements for large-scale manufacturing, and insufficient stability for long-term storage [11]. Overcoming these limitations is crucial for the successful commercialization of exosome-based therapeutics.

Although cell-based drug delivery systems, including macrophages, stem cells, and exosomes, offer promising avenues for targeting brain tumors, their ability to penetrate an intact BBB or even a partially compromised BTB remains limited. Rather than facilitating BBB traversal per se, these systems exploit intrinsic tumor-tropic properties to enhance drug accumulation within tumor tissue once the barrier has been bypassed. Effective strategies will therefore require complementary approaches to modulate BBB or BTB permeability, or the use of engineered carriers capable of active transcytosis. Continued innovation at the interface of cellular engineering and nanotechnology will be critical to fully unlocking the potential of cell-based therapies in neuro-oncology.

Crucially, quantitative data on the proportion of the administered cell-based therapy dose that reaches the tumor (% of injected dose) remain critically lacking, despite their importance for evaluating delivery efficiency. This absence highlights a key knowledge gap in the field, underscoring the need for comprehensive studies that focus on in vivo delivery metrics to optimize cell-mediated brain tumor therapies.

### 3.43. Bridging Therapy: Integrating Neurosurgical Techniques with Pharmacological Strategies

Despite significant advances in pharmacological strategies designed to bypass or traverse the BBB and BTB, therapeutic delivery to brain tumors remains suboptimal. In many cases, these approaches fail to achieve therapeutically meaningful concentrations across the heterogeneous and hostile microenvironment of brain tumors. This is because, once within the CNS, therapeutic agents must contend with the elevated ISF pressure and a dense ECM, which impede drug diffusion, distribution, and cellular uptake. These limitations are particularly pronounced in therapy-resistant niches such as pseudopalisading glioma cells and infiltrative tumor margins.

This therapeutic impasse necessitates a bridging strategy: the integration of pharmacological innovation with interventional and neurosurgical delivery techniques. Approaches such as osmotic BBB disruption (OSBBBD) via intra-arterial (IA) infusion, focused ultrasound (FUS), and convection-enhanced delivery (CED)—which bypasses the BBB through direct parenchymal infusion—have advanced considerably and now offer the potential to improve local drug concentrations. Moreover, microsurgical resection, a cornerstone of brain tumor management, transiently disrupts the BBB and BTB, creating a window of opportunity for targeted therapeutic intervention. In this context, theranostic platforms can be engineered to exploit the altered microenvironment of the resection cavity. In the sections that follow, we explore the rationale and emerging evidence for these hybrid strategies, underscoring their synergistic potential to reshape therapeutic paradigms in neuro-oncology. A comprehensive technical discussion of each method lies beyond the scope of this review and can be found elsewhere [454,455].

### 3.44. Osmotic Blood–Brain Barrier Disruption with Intra-Arterial Chemotherapy

OBBBD, most commonly achieved via IA infusion of 25% mannitol (10 mL; 1.37 mmol/L), remains one of the most established techniques for enhancing drug accumulation in brain tumors [454,455,456]. Supported by decades of preclinical and clinical data, OBBBD is typically performed alongside IA chemotherapy to bypass the first-pass effect, thereby increasing both the peak plasma concentration and AUC of the administered drug [456]. Additionally, IA delivery improves local perfusion and can counteract elevated IFP, promoting better drug distribution within the tumor [454,455]. OBBBD/IA’s potential to improve drug tumor concentrations is considerable. OBBBD with IA delivery increased carboplatin deposition in the brain approximately 17-fold compared to IA alone, and provided a 320-fold increase compared to intravenous (IV) administration [457]. Moreover, a single IA dose of bevacizumab (15 mg/kg) following OBBBD produced comparable or superior progression-free survival relative to biweekly IV dosing (10 mg/kg) [458]. Importantly, by extending tumor exposure to high drug concentrations, OBBBD facilitates more uniform distribution across the entire cerebral vascular territory, including the infiltrative tumor margins [454,455]. This helps overcome the “sink phenomenon,” where chemotherapeutic agents accumulate predominantly in necrotic tumor cores, leading to the underdosing of viable, proliferative regions [456]. Consequently, OBBBD enhances both the depth and breadth of drug penetration into clinically relevant tumor areas. Importantly, by modulating mannitol dose, infusion rate, and catheter positioning, the extent of OBBBD can be tailored to clinical needs. Placement in the proximal internal carotid artery or the distal V2 segment of the dominant vertebral artery enables widespread BBB disruption, improving drug delivery to both tumor tissue and adjacent brain parenchyma [456]. This is especially valuable in treating infiltrative gliomas or multifocal lesions, such as metastases from breast or lung cancer. In contrast, well-localized tumors may benefit from the selective catheterization of small feeding arteries to achieve focused BBB disruption and limit off-target exposure [459].

Although the concept of OBBBD with IA delivery originated in the latter half of the 20th century, interest in this approach has been renewed by recent technological advancements. Innovations such as dual-lumen balloon microcatheters, which allow for controlled cerebral perfusion, and state-of-the-art angiographic tools enabling precise targeting of distal, tumor-feeding vessels have significantly enhanced the feasibility and safety of this method.

Clinical studies consistently demonstrate the safety of OBBBD combined with IA delivery [456,460,461,462]. Although transient side effects such as cerebral edema, aphasia, or hemiparesis may occur due to rapid fluid shifts or neurotoxicity, these risks can be significantly mitigated through optimal catheter placement, real-time imaging, flow-arrest techniques, and pulsatile injection protocols. In a retrospective analysis of 3837 procedures in patients with primary or metastatic brain tumors, major and minor complication rates were reported at 0.70% and 7.53%, respectively [460]. The incidence of stroke was 1.3% for OBBBD/IA therapy and 0.5% for IA delivery alone [462]. To date, most clinical experience has involved traditional cytotoxic agents [454,455], underscoring the need for studies evaluating next-generation therapies—including molecularly targeted agents, immunotherapies, gene therapies, and NP-based drug systems. Early-phase clinical trials are currently investigating OBBBD/IA administration of cetuximab in newly diagnosed GBM (NCT02861898) and bevacizumab in recurrent GBM (NCT02285959). A particularly promising strategy involves combining OBBBD and IA delivery with cell-based platforms that exhibit intrinsic tumor tropism. However, to date, only one clinical trial (NCT03896568) has explored catheter-based IA delivery of MSCs loaded with the oncolytic virus Delta-24-RGD in recurrent GBM—without incorporating OBBBD [463]. Given that OBBBD is a safe and effective method for enhancing tumor drug concentrations when performed by experienced teams, there is a clear and pressing need for well-designed clinical trials to evaluate emerging therapeutic modalities [464]. Expanding the clinical pipeline to incorporate OBBBD-augmented delivery of novel agents will be essential to translating these promising strategies into meaningful survival benefits for patients with malignant brain tumors.

### 3.45. Convection-Enhanced Delivery

CED is a neurosurgical technique that utilizes stereotactically placed catheters connected to infusion pumps to generate continuous positive pressure, enabling direct delivery of drugs to large, targeted areas with predominantly spherical distribution [465]. While the primary driving force behind CED is bulk flow, a secondary concentration gradient ensures further drug dispersion into surrounding tissues even after infusion ceases. As a result, CED achieves high intratumoral drug concentrations not only within radiographically defined tumor regions but also in infiltrated peritumoral brain tissue—making it significantly more effective than passive-diffusion-based approaches such as intracerebral implants (Gliadel^®^ wafers) or intratumoral injections [466]. Whereas single-injection techniques generally achieve therapeutic penetration of up to 5 mm from the catheter tip, CED extends distribution up to 6 cm, leading to a 4000-fold increase in the volume of distribution (Vd) [454,467]. This enhanced distribution helps overcome issues related to high intratumoral IFPs [468].

Numerous clinical trials have established CED as a safe and viable strategy to bypass the BBB and deliver a broad spectrum of agents—including chemotherapeutics [469], conjugated toxins [470], liposomes [471], and viruses [472]—to large CNS regions in GBM patients. Although tested in a limited number of cases, CED-delivered immunotherapies, particularly oncolytic viruses and TGF-β2 inhibitors, have shown notable activity [465]. However, sustained clinical responses remain rare, reflecting the profound immunosuppression characteristic of gliomas [465]. CED has also been studied in pediatric patients with DIPG; a recent Phase I trial demonstrated the safety of IL13-Pseudomonas toxin infusion into the brainstem using this method [473]. Both preclinical and clinical analyses have shown that CED achieves high concentrations of therapeutic agents in regional brain parenchyma and adjacent white matter tracts, with minimal efflux when the BBB remains intact [474,475,476].

Nevertheless, clinical trials have revealed several challenges: drug efflux, catheter-induced trauma, uneven drug distribution, poor penetration into the invasive margins of diffuse gliomas, and the lack of real-time visualization of drug delivery [467]. While catheter design improvements are being pursued to address mechanical issues, multifunctional nanoscale materials offer promising solutions to the remaining limitations. These nanocarriers enable real-time imaging, controlled drug release, optimized size and surface properties for enhanced tissue distribution, and targeted delivery through ligand conjugation. One notable system, NPCP-CTX—a PEGylated NP with an iron oxide core for MRI visualization, a chitosan coating for improved DNA interaction, and chlorotoxin (CTX) for GBM targeting—demonstrated a 3.4-fold increase in Vd 24 h post-CED compared to non-targeted NPCP, and a 10-fold improvement over similar systems [477]. The distribution was sustained for over 48 h, and the system provided T2-weighted MRI contrast for real-time monitoring. Furthermore, NPCP-CTX preferentially localized to GBM cell nuclei, supporting its potential for highly selective therapeutic delivery. Similarly, a recent clinical trial involving 18 patients with high-grade glioma demonstrated the feasibility of the CED of liposomal irinotecan under real-time MRI guidance, reporting favorable safety and improved survival outcomes compared to historical controls. These findings jointly strengthen the rationale for further investigation of CED combined with multifunctional nanotechnologies to maximize clinical benefit in GBM treatment [471].

### 3.46. Focused Ultrasound

High-frequency FUS is primarily used for the thermal ablation of targeted tissues, while low-frequency FUS facilitates mechanical disruption of the BBB, presenting a promising strategy for enhancing drug delivery to the brain. This approach has gained significant attention in neuro-oncology, leading to the development of several devices—such as SonoCloud (CarThera Inc., Lyon, France), ExAblate (INSIGHTEC Inc., Tirat Carmel, Israel), and NaviFUS (NaviFUS Inc., Taipei, Taiwan)—which have advanced to clinical trials [478,479,480,481,482]. All three technologies rely on the same fundamental mechanism for transient BBB/BTB opening: ultrasound waves that target intravenously injected microbubbles. These microbubbles oscillate in response to the alternating rarefaction and compression phases of the pressure wave, exerting mechanical stress on bECs and inducing the opening of TJs [483]. Recent studies examining the acute response of human bECs to ultrasound-mediated BBB disruption have revealed transcriptional changes in genes related to neurovascular barrier function, including those involved in the BM, EC cytoskeleton, junction complexes, caveolae transcytosis, and solute transporters [484]. The ultrastructural changes observed included a reduction in luminal caveolae, the emergence of cytoplasmic vacuoles, and the disruption of the BM and TJs [484].

Notably, there are differences in the design of these technologies: SonoCloud requires surgical implantation into the skull, whereas ExAblate and NaviFUS are non-invasive. ExAblate utilizes a transducer mounted in a helmet and requires the procedure to be performed in an MRI suite, with stereotactic fixation of the patient’s head. In contrast, NaviFUS is a phased-array system that integrates with clinically available neuronavigation systems for precise targeting. Clinical trials have confirmed that FUS enables safe and transient BBB/BTB opening in localized brain regions, significantly increasing the concentrations of systemically administered drugs, such as trastuzumab, carboplatin, TMZ, liposomal DOX, and albumin-bound PTX, within brain tumors [483]. A recent Phase I trial in recurrent GBM patients showed a 3.7-fold increase in PTX and a 5.9-fold increase in carboplatin concentrations in brain tissue [485]. Besides transiently disrupting the BBB and BTB, recent preclinical evidence shows that FUS significantly enhances intratumoral interstitial transport, thereby improving the convective distribution of therapeutics throughout the tumor matrix [486]. FUS application led to a two-fold increase in mean ISF velocity within brain tumors, thereby augmenting convective drug movement through the tumor matrix. Additionally, FUS induced a marked reorientation of fluid flow—altering directionality by an average of 70° to 80°—suggesting a substantial remodeling of interstitial dynamics. This mechanical modulation translated into over a 100% increase in the distribution of locally administered brain-penetrating NPs, highlighting FUS as a potent enhancer of both barrier permeability and intratumoral drug dispersion [486].

While the potential of FUS to improve tumor drug concentrations has been validated, concerns about off-target delivery and potential damage to healthy brain tissue persist. To address these concerns, there has been increasing interest in combining FUS with NPs decorated with ligands targeting specific tumor cell markers to enhance the precision of drug delivery. For example, researchers have developed a liposomal complex with the NGR peptide, which binds to the CD13 receptor overexpressed in glioma cells and bECs, enabling targeted delivery of gene therapy. Separately, DOX-loaded liposomes linked to human atherosclerotic plaque-specific peptide-1 (AP-1) have been developed to target interleukin-4 receptors (IL-4R) upregulated on brain tumor cells [487].

FUS is a unique tool that not only transiently disrupts the BBB and BTB but also increases ISF velocity and reorients fluid flow, enabling the deeper and more uniform penetration of therapeutics into the tumor matrix—a key limitation of systemic drug delivery. When combined with NPs functionalized with tumor-specific ligands [488], this dual-action approach integrates molecular precision with spatial control. Rationally designed FUS–NP platforms offer a promising strategy to overcome both vascular and interstitial barriers, advancing targeted drug delivery in neuro-oncology.

### 3.47. Microsurgery

Surgical removal of the tumor bulk is the primary treatment for GBM. However, due to its infiltrative nature, highly tumorigenic GSCs extend beyond the visible tumor bulk into surrounding healthy brain tissue where they are shielded by an intact BBB. As complete excision is virtually impossible, residual self-renewing GSCs drive GBM recurrence at the surgical margin. Notably, surgery is known to transiently disrupt the BBB [489]. A recent preclinical study described a biphasic pattern of BBB disruption at the resection cavity: the first phase occurs immediately postsurgery, while a second phase emerges two to three days later, likely due to inflammation and tissue remodeling [489]. During these windows, fluorescently labeled lipid NPs (LNPs) selectively accumulated at the resection margin while being excluded from brain regions with an intact BBB. Confocal microscopy confirmed LNP extravasation into the parenchyma and uptake by glial cells associated with residual tumor. Exploiting this transient permeability, DOX-loaded LNPs administered during peak BBB disruption completely inhibited tumor recurrence after a single dose. In contrast, delayed administration—consistent with standard postoperative timelines—failed to achieve comparable efficacy. Moreover, free DOX, lacking the NP carrier, was therapeutically ineffective, underscoring the critical role of nanocarrier-based delivery [489].

This transient post-resection BBB opening is often overlooked in experimental GBM treatment paradigms but offers a unique opportunity for targeted drug delivery. To harness this window, researchers developed a platelet-membrane-derived vesicle (PMV) system engineered into neurotransmitter-mimicking nanovesicles (PMVS-P) loaded with the anti-GSC agent salinomycin (SAL) and coated with polydopamine (PDA). In preclinical models, the nanocarrier selectively localized to the resection cavity due to the platelets’ innate injury-homing behavior, while the PDA surface enabled GSC targeting via dopamine-D2-receptor-mediated recognition, leading to effective inhibition of tumor recurrence [490]. Beyond increased permeability, the postsurgical brain also releases inflammatory cytokines such as interleukin-8 (IL-8) and tumor necrosis factor alpha (TNF-α), which recruit immune cells to the resection margin. This microenvironment enables neutrophils (NEs) to serve as endogenous drug carriers. In a complementary study, NEs were loaded with PTX-encapsulated liposomes and directed to inflamed postsurgical tissue [491]. The inflammatory milieu triggered localized release of PTX from NEs, significantly reducing tumor recurrence in murine models. However, residual tumor cell islands were still detected in the surrounding brain, likely due to PTX resistance or the incomplete targeting of infiltrative cells. These findings emphasize the need to develop precision drug systems capable of targeting GSCs within the infiltrative tumor margin—a major obstacle to durable GBM control.

### 3.48. Conclusions and Future Directions

Achieving pharmacologically meaningful drug concentrations within brain tumors remains one of the most significant hurdles in neuro-oncology. Despite considerable progress in understanding the molecular biology of brain tumors and the development of tumor-specific therapeutics, overcoming the physiological barriers presented by the BBB and BTB remains a critical bottleneck. While a variety of strategies to enhance drug penetration across the BBB and BTB have been explored, only a few have reached clinical trial stages, and none have yet been widely adopted. Efforts aimed at chemically modulating the BBB through receptor-mediated TJ disruption via the activation of receptors have thus far failed to yield clinically meaningful improvements in drug accumulation. Clinical trials have not demonstrated consistent increases in intratumoral drug concentrations or survival benefit, largely due to nonspecific receptor distribution, short-lived permeability changes, and variability in patient responses. Research is now shifting focus toward identifying new agonists and receptors that may offer more specificity, but challenges related to off-target effects and safety still present significant concerns.

Efflux transporters, particularly P-gp/ABCB1, pose a major barrier to therapeutic drug accumulation in brain tumors by actively extruding a broad spectrum of chemotherapeutic agents from bECs. Although the co-administration of efflux pump inhibitors has been proposed to enhance drug retention within the CNS, clinical trials have thus far failed to demonstrate benefit. This is largely attributable to the difficulty in achieving therapeutic inhibitor concentrations at the BBB without eliciting systemic toxicity. Furthermore, the ubiquitous expression of efflux transporters in peripheral organs increases the risk of off-target effects and pharmacokinetic interactions, limiting the clinical applicability of current inhibitors. As a result, selective modulation of efflux transporters at the neurovascular interface remains an unmet need. Future efforts must focus on developing localized or transient inhibition strategies—potentially via NP-mediated delivery or inducible systems—to overcome this barrier without compromising systemic safety.

Developing antitumor agents capable of passively diffusing across the BBB has proven largely unfeasible due to stringent physicochemical constraints. As therapeutic molecules become increasingly complex and larger in size, it is evident that active transport mechanisms are essential for efficient CNS drug delivery. Similarly, passive NP-based strategies relying on the EPR effect demonstrated limited success in the brain, highlighting the need to pivot toward active targeting approaches that utilize ligands to engage specific transcytotic pathways.

CMT-based drug delivery systems, while theoretically appealing, are often too large to exploit narrow transmembrane channels and rely primarily on RMT for brain entry. However, the broad peripheral expression of many RMT targets increases the risk of systemic off-target effects and limits therapeutic selectivity. Dual-targeting strategies that combine BBB engagement with tumor-specific ligands have shown promise in preclinical models, but face translational barriers, including system complexity, suboptimal pharmacokinetics, and unpredictable biodistribution. AMT ligands, meanwhile, are hindered by a low affinity for endothelial targets and are often sequestered within bECs, reducing effective drug delivery. Additionally, AMT-based systems have been associated with neurotoxicity, further limiting their clinical applicability.

Cell-based delivery platforms have demonstrated potential for crossing the BTB and delivering payloads deep into the tumor parenchyma. However, due to their poor penetration across the BBB under physiological conditions, adjunctive strategies such as FUS, OBBBD, or CED are required to enhance drug concentrations within tumors.

Much of our current understanding of BBB and BTB biology—and many of the strategies proposed to overcome these barriers—derives from preclinical studies in rodent models. While these models have been invaluable for uncovering mechanistic principles, they often fail to replicate the anatomical, vascular, and immunological complexity of human brain tumors, limiting their translational relevance. Future research should prioritize validation in more clinically relevant systems, including patient-derived xenografts, human organoids, and early-phase clinical studies.

The convergence of pharmacological and neurosurgical approaches offers a promising framework for overcoming the limitations of standalone strategies. Integrating cell-based carriers and nanoscale delivery systems with FUS, OBBBD, or CED can enhance both the depth and uniformity of drug distribution within infiltrative brain tumors. While early clinical data suggest feasibility, optimization of these combinations remains an area requiring urgent refinement.

Clinical trials must go beyond traditional endpoints such as overall and progression-free survival. Pharmacokinetic assessments—measuring the intratumoral drug concentration, volume of distribution, and duration of exposure—are essential to understanding the true therapeutic impact of delivery systems. Head-to-head comparisons of emerging platforms will be key to identifying the most effective regimens tailored to specific tumor types, drug classes, and delivery routes.

In conclusion, meaningful advances in brain tumor therapeutics will depend on a multidisciplinary, mechanistically informed approach that unites novel pharmacological strategies with precise, image-guided delivery techniques. Bridging the gap between molecular innovation and clinical applicability will require rigorous translational studies and a sustained commitment to overcoming the formidable barriers posed by the BBB and BTB.

### 3.49. Limitations

This scoping review is limited by the heterogeneity of the available literature on BBB and BTB modulation, including variability in experimental design, sample characteristics, and study size. The section on pharmacological strategies is particularly constrained by the predominance of preclinical data, as relatively few approaches have advanced to clinical trials. Many candidate therapies have been evaluated under idealized laboratory conditions that may not adequately reflect the complexity of the human brain TME. Additionally, the absence of standardized protocols for assessing drug concentration, distribution volume, and pharmacokinetics across studies limited the ability to compare outcomes directly. The inclusion of a broad range of study designs further precluded quantitative synthesis. Lastly, the evidence base may be affected by publication bias, as studies with negative or inconclusive results are less likely to be published, potentially overstating the effectiveness of certain pharmacological strategies.
